# Supplementary material for: Proteomic profiling reveals CDK6 upregulation as a targetable resistance mechanism for lenalidomide in multiple myeloma
Source: Nat Commun. 2022 Feb 23;13:1009. doi: 10.1038/s41467-022-28515-1 (PMC8866544; doi:10.1038/s41467-022-28515-1)
Supplement: Supplementary file 1 — Supplementary Information [file 41467_2022_28515_MOESM1_ESM.pdf]

Supplementary Figure 1

Analysis	Patient #	Sample Time point	Ig-Type	FISH Diagnosis	Treatment	Time between samples (months)	FISH Relapse
Proteomics, RNA seq, WES ID #16	1	Diagnosis + Relapse	IgA lambda	t(14;16), +17p13, penta 1q21, +1p32, tetra 9q34	Len / Dex	28	t(14;16), +17p13, tetra 1q21, +1p32, +9q34, del13q14
Proteomics, RNA seq	2	Diagnosis + Relapse	IgG kappa	+9q34, +1q21	Len / Dox / Dex<br>HD-Mel+ Auto-SCT<br>Len maintenance	24	+9q34, tetra 1q21
Proteomics, RNA seq, WES ID #14	3	Diagnosis + Relapse	kappa	t(11;14)	Len / Dex/ HD-Mel+ Auto-SCT<br>Len maintenance	19	t(11;14), +1q21
Proteomics, RNA seq, WES ID #9	4	Diagnosis + Relapse	lambda	t(11;14)	Len / Dex	33	t(11;14)
Proteomics, RNA seq, WES ID #1	5	Diagnosis+ Relapse	IgA kappa	t(4;14) , +1q21, del13q14	Mel / Pred<br>Len / Dex<br>Bort / Dex<br>Pom / Dex/ HD-Mel+ Auto-SCT	46	t(4;14) , +1q21, del13q14
Immunoblot	6	Diagnosis	lambda	t(4;14), +1q21, del13q			
Immunoblot	7	Diagnosis	IgG lambda	t(4;14), del 13q			
Immunoblot	8	Diagnosis	lambda	t(11;14)			
Immunoblot	9	Diagnosis	kappa	t(11;14), del17p13, del13q14			
Immunoblot	10	Relapse	IgG lambda		B/ HD-Mel+ Auto-SCT/ DRD		t(4;14), del13q14, +1q21
Immunoblot	11	Relapse	IgA kappa		BCD/ HD-Mel+auto<br>Len / Dex		+9q34.2. +1q21, +17p13.1
Immunoblot	12	Relapse	IgA kappa		Carf / Mel / Pred<br>Len / Pred		+9q34
Immunoblot	13	Relapse of #8	lambda		BCD/ HD-Mel+ Auto-SCT<br>Len / Dex<br>Carf / Dex/ HD-Mel+ Auto-SCT<br>Len / Bort / Dex<br>Dara<br>Pom / Dex		t(11;14)
Immunoblot	14	Relapse	IgA lambda		Len / Bort / Dex<br>2x HD-Mel+ Auto-SCT		t(14;16), tetra 1q, del13q,
Immunoblot	15	Relapse of #7	IgG lambda		ID/ 2x HD-Mel+ Auto-SCT		t(4;14), del 13q
Immunoblot	16	Relapse	IgG lambda		BCD/ 2x HD-Mel+ Auto-SCT/B<br>DRD		t(4;14), +1q21, del13q,
Immunoblot	17	Relapse	IgA lambda		Bort / Thal / Dex / Cis / Dox / CP / E		tetra 1q21, +13q14, del17p, t(14;16)
Immunoblot	18	Relapse	IgG lambda		BCD/ HD-Mel+ Auto-SCT/B<br>Len maintenance<br>Vor / Dex / Dara		+9q34

BCD= bortezomib/ cyclophosphamide/ dexamethasone; Bort = bortezomib; Carf = carfilzomib; Cis = cisplatin; CP = cyclophosphamide; Dara = daratumumab; Dex = dexamethasone; Dox = doxorubicin; DRD= daratumumab/ lenalidomide/ dexamethasone; E = etoposide; HD-Mel+ Auto-SCT = High dose melphalan with autologous stem cell transplantation; ID = idarubicune/ dexamethasone; Len = lenalidomide; Mel = melphalan; Pom = pomalidomide; Pred = prednisone; Thal = thalidomide; Vor = Vorinostat.

Supplementary Figure 1. Patient characteristics. WES ID is referenced to Bohl *et al*. 2021.

Supplementary Figure 2

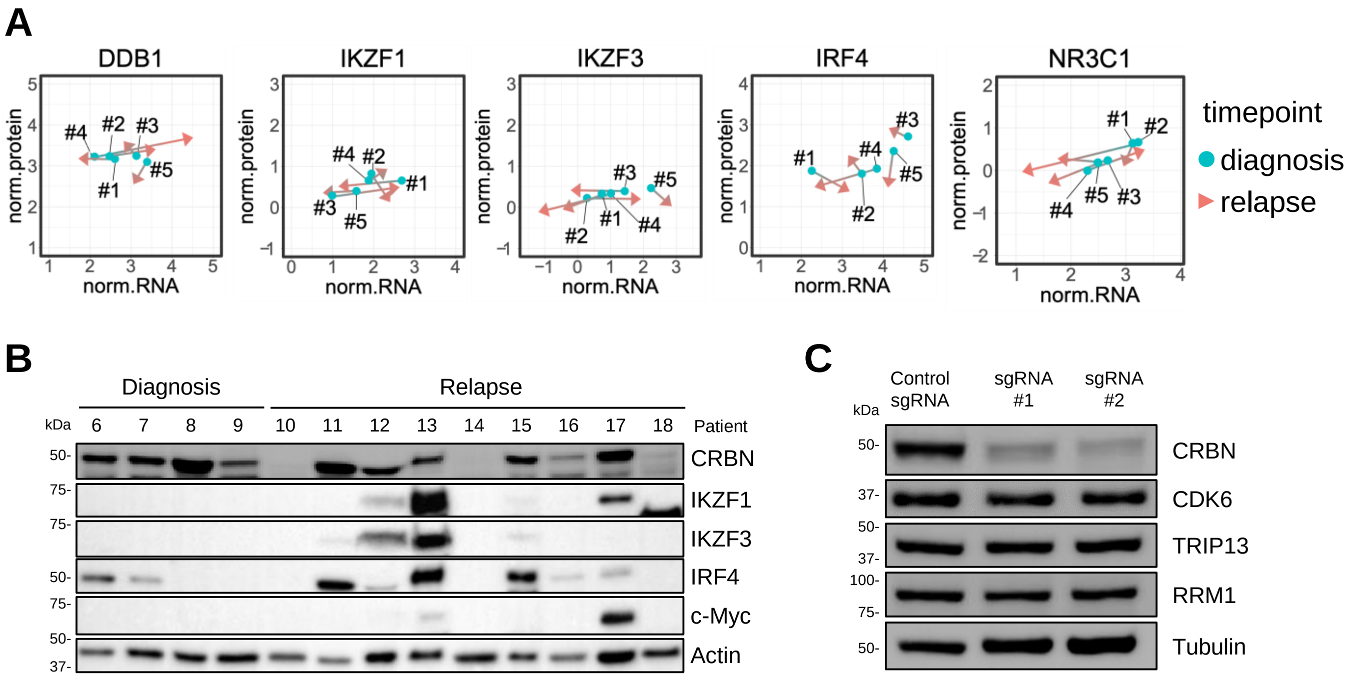

Supplementary Figure 2. Protein and RNA expression levels of proteins involved in the mechanism of thalidomide analogs in multiple myeloma. (A) Median normalized protein intensities (log2 TMT intensities) of DDB1, IKZF1, IKZF3, IRF4 and NR3C1 in all 10 samples were plotted against their respective normalized RNA expression levels (log2 TPM values). Samples from the same patient are connected. **(B)** Western blot of additional patient cohort for expression of IMiD-related proteins. (N=13 patient samples) **(C)** CRISPR/Cas9-mediated knockout of CRBN and protein levels of CDK6, TRIP13, and RRM1 detected by western blot. (N=3 biologically independent replicates)

Supplementary Figure 3

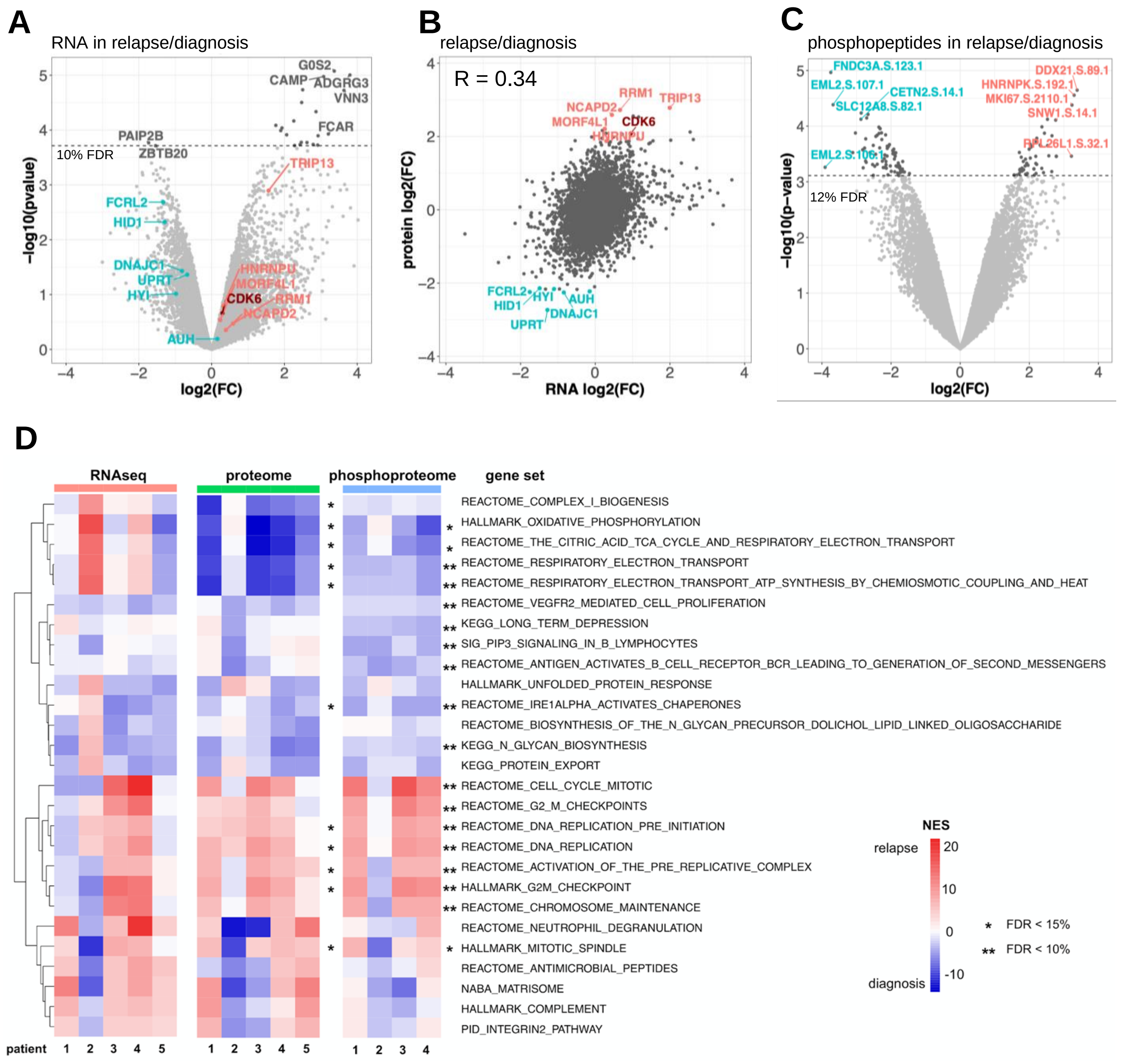

Supplementary Figure 3. RNA sequencing, phosphoproteomic analysis, and top deregulated gene signatures in paired multiple myeloma patient samples. (A) RNA expression changes at relapse/diagnosis were determined for each patient and analyzed with a two-sided moderated 1-sample t-test. Average log2(fold change) of each transcript is plotted against its –log10(p-value). The corresponding transcripts of the 6 most up and down regulated proteins are highlighted in color. (B) Correlation of average fold changes (log2(FC)) of protein and RNA expression levels. (C) Phosphopeptide abundance level changes at relapse/diagnosis were determined for each patient and analyzed with a two-sided moderated 1-sample t-test. Average log2(fold change) of each phosphopeptide is plotted against its –log10(p-value). (D) Single sample gene set enrichment analysis (ssGSEA) of relapse/diagnosis ratios in each patient. Heatmap displays the normalized enrichment scores (NES) of the most regulated gene sets in RNAseq, global proteome and phoshoproteome datasets for each patient. Significance of terms in each dataset is indicated with stars. Significance was determined with a one-sample moderated t-test.

Supplementary Figure 4

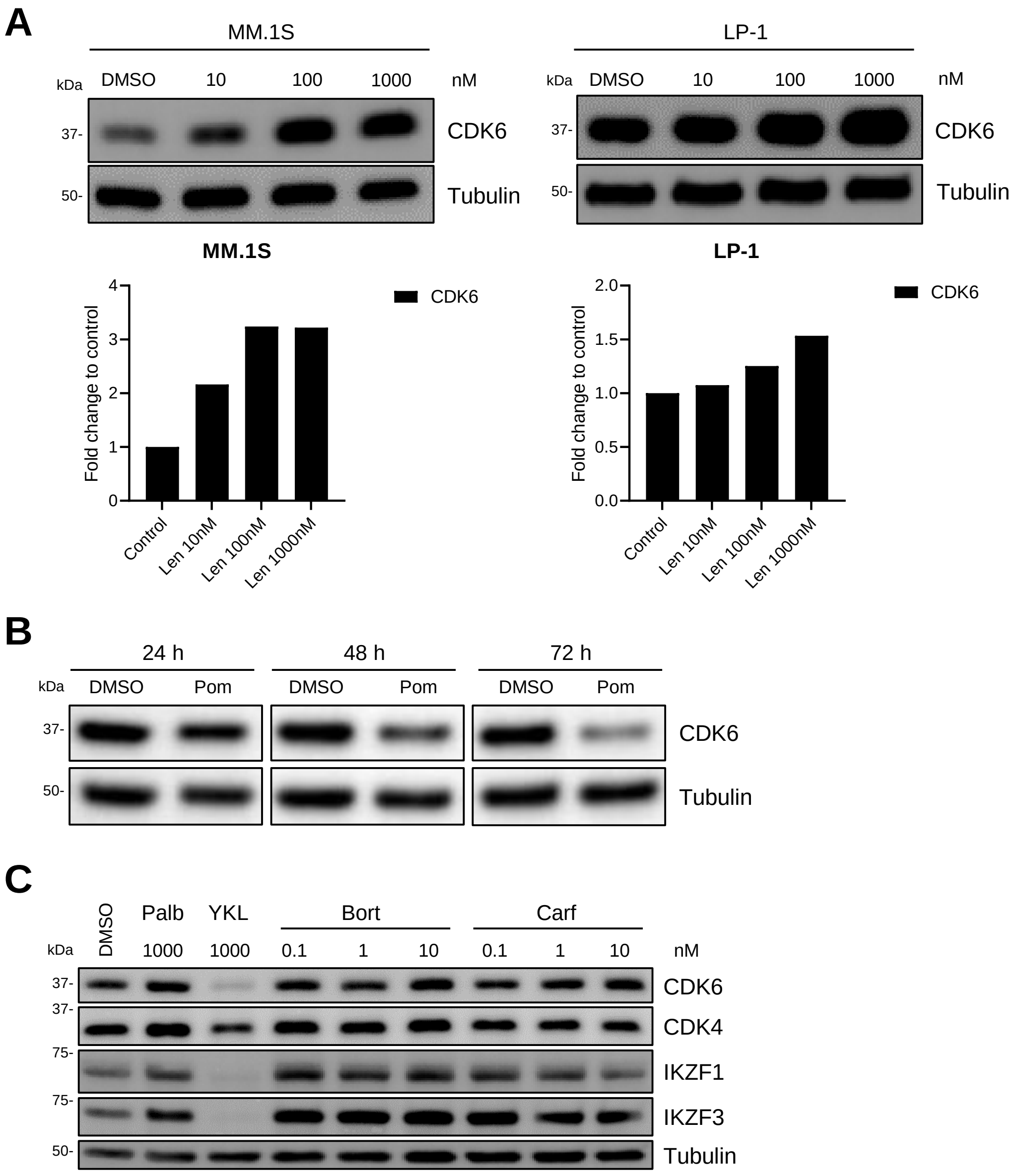

Supplementary Figure 4. CDK6 protein levels in multiple myeloma cells treated with IMiDs and proteasome inhibitors. Multiple myeloma cells MM.1S and LP-1 were cultured in the presence of respective concentrations of lenalidomide (len) to induce drug-resistance. (A) CDK6 protein levels of lenalidomide-resistant (lenR) cells and respective quantification. CDK6 protein levels were normalized to respective loading controls and to treatment control. (B) Treatment of MM.1S cells with 1 µM of pomalidomide (pom) for 24, 48, or 72 h. (C) Treatment of MM.1S with palbociclib (palb), YKL-06-102 (YKL), and proteasome inhibitors bortezomib (bort) and carfilzomib (carf) at respective concentrations for 4 h. (N=3 biologically independent replicates)

Supplementary Figure 5

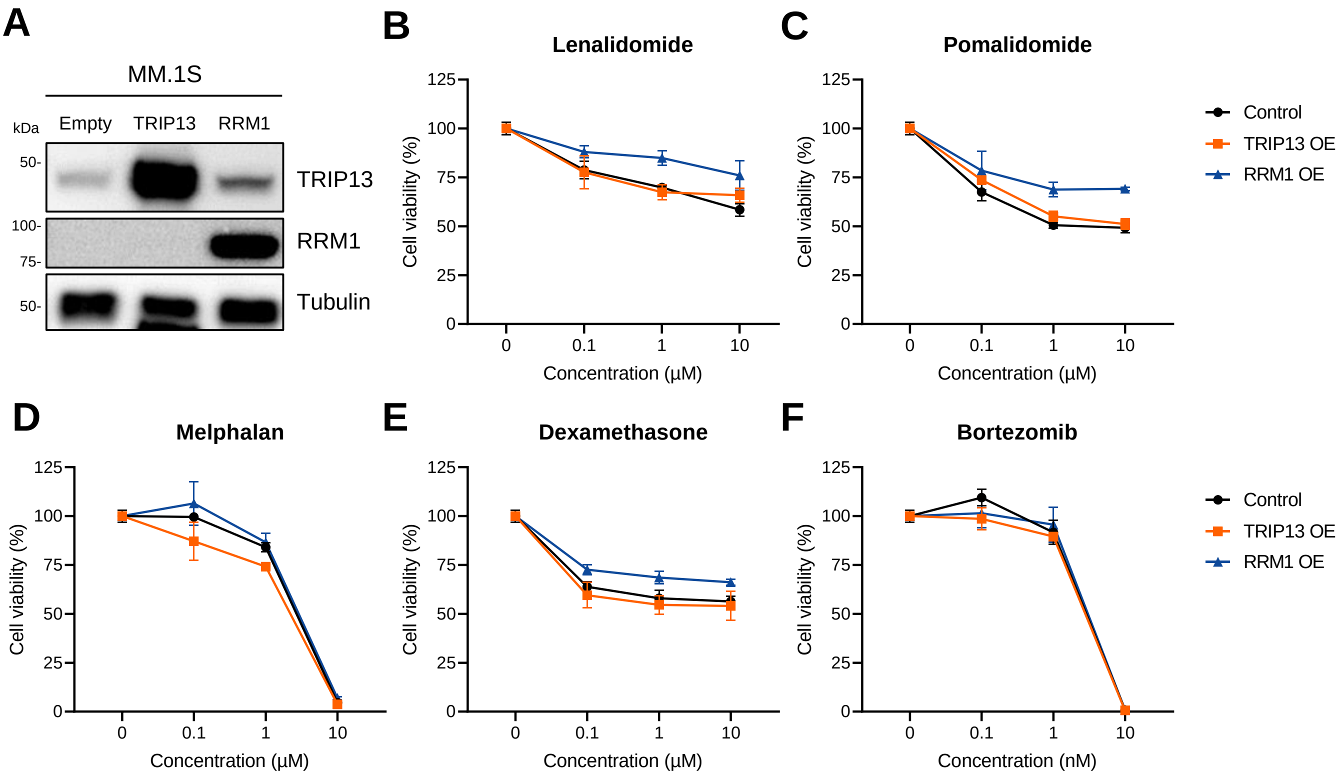

Supplementary Figure 5. Effects of elevated TRIP13 or RRM1 protein expression on cell viability in response to multiple myeloma therapies. (A) Overexpression of TRIP13 and RRM1 in MM.1S cells using retroviral transduction confirmed through western blot analysis. (B) Cell viability of RRM1- and TRIP13-overexpressing MM.1S cells after subjected to 96 h treatment with lenalidomide, (C) pomalidomide, (D) melphalan, (E) dexamethasone, and (F) bortezomib. (N=3 biologically independent replicates) Control denotes empty vector. Cell viability is normalized to respective DMSO conditions. Data represent the mean ±SD of biological triplicates.

Supplementary Figure 6

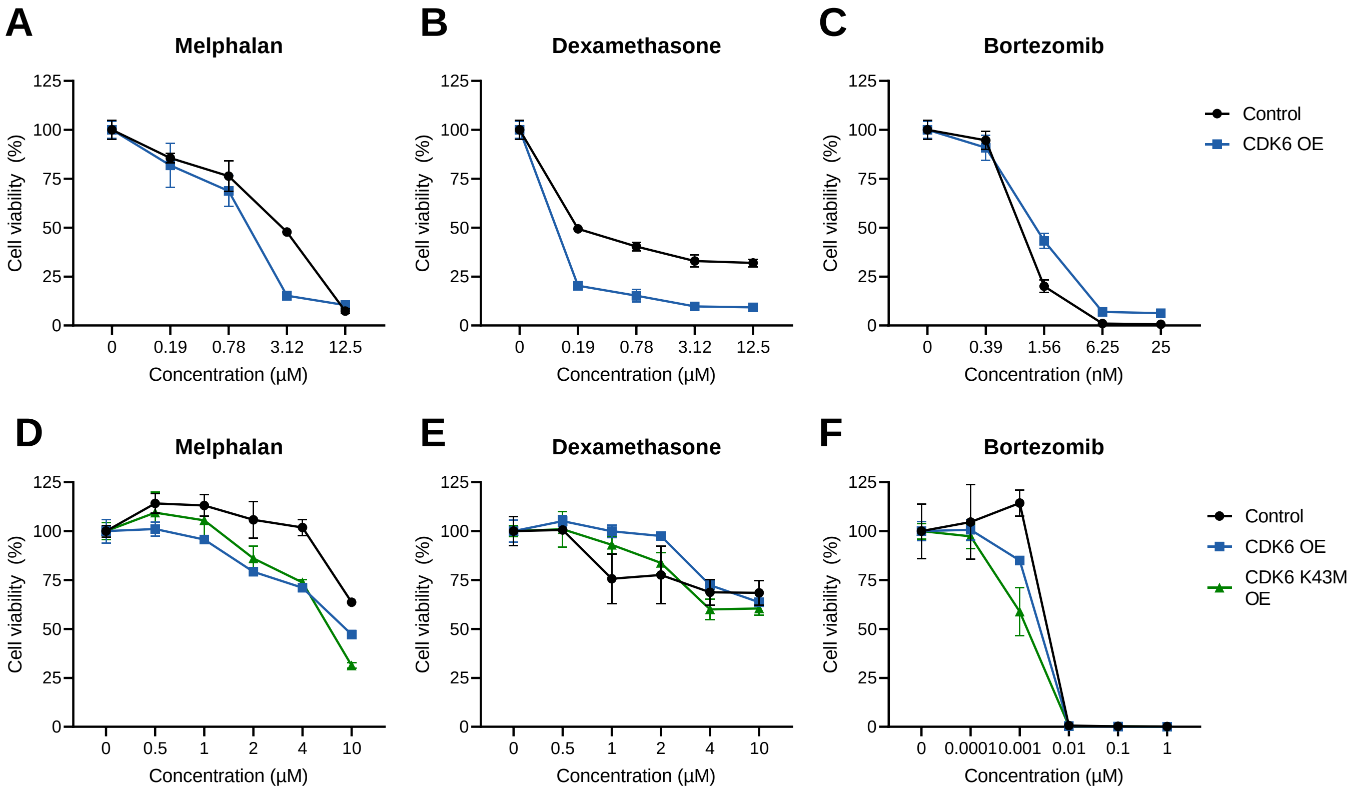

Supplementary Figure 6. Effects of CDK6 overexpression in response to multiple myeloma therapies. (A-C) Cell viability of 96 h treatment with melphalan, dexamethasone and bortezomib in MM.1S cells overexpressing CDK6 WT and in (D-F) OPM2 cells overexpressing CDK6 WT and CDK6 K43M mutant. (N=3 biologically independent replicates) Control denotes empty vector. Cell viability is normalized to respective DMSO conditions. Data represent the mean ±SD of biological triplicates.

Supplementary Figure 7

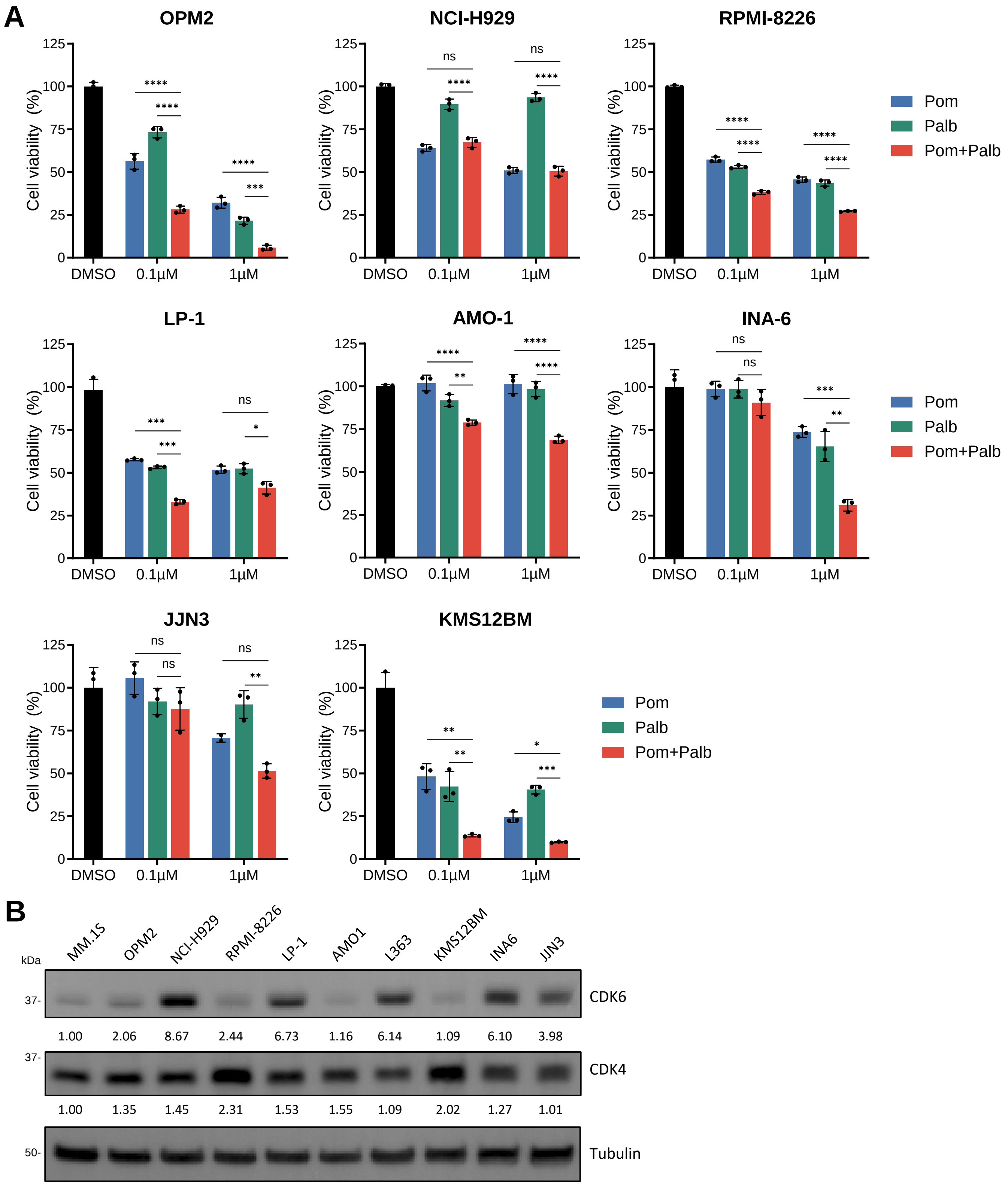

Supplementary Figure 7. CDK6 inhibition by palbociclib sensitizes multiple myeloma cells to IMiDs. (A) Cell viability of IMiD-sensitive and -resistant multiple myeloma cell lines when treated for 96 h with pomalidomide (pom), palbociclib (palb), and in combination (pom+palb). (N=3 biologically independent replicates) (B) Endogenous levels of CDK6 across all multiple myeloma cell lines. Cell viability is normalized to respective DMSO conditions. Data represent the mean ±SD of biological triplicates. One-way ANOVA is applied. P values are displayed as follows: n.s. = P > 0.05; * = P ≤ 0.05; ** = P ≤ 0.01; *** = P ≤ 0.001; **** = P ≤ 0.0001. Quantification of CDK6 and CDK4 is normalized to tubulin and to MM.1S.

Supplementary Figure 8

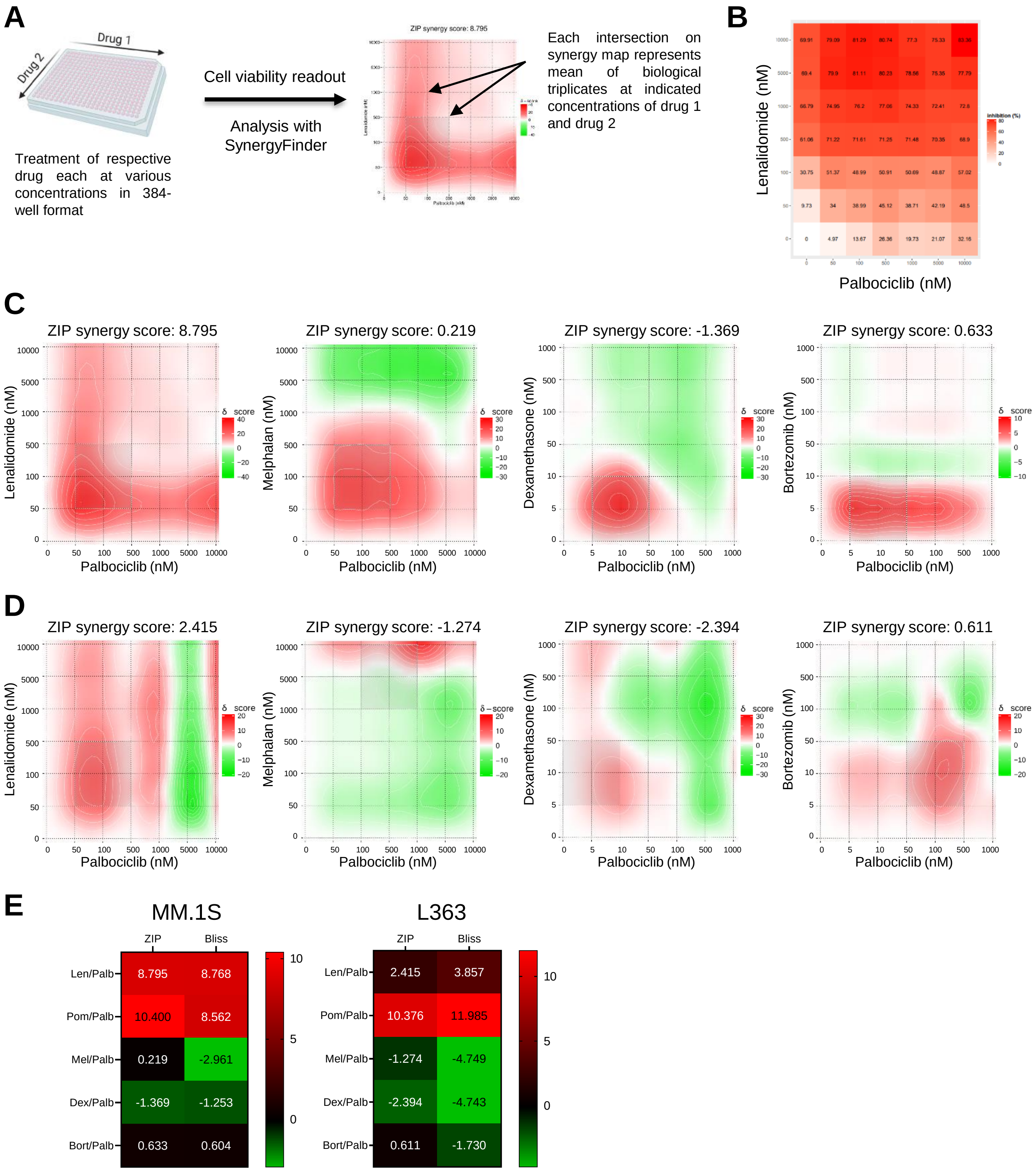

Supplementary Figure 8. Drug synergy analyses of palbociclib with multiple myeloma drug treatments. (A) Workflow for obtaining synergy plots. (B) MM.1S cell viability upon combination treatment of palbociclib with lenalidomide. (C) Synergy plots of 96 h treatment of palbociclib in combination with lenalidomide, melphalan, dexamethasone, and bortezomib in MM.1S and (D) L363 cells. (E) Heatmap of synergy scores of combination treatments with palbociclib. Synergy plots, ZIP score and Bliss score calculations were generated and performed with SynergyFinder.[75] Data is normalized to respective DMSO conditions and represent the mean of biological triplicates.

Supplementary Figure 9

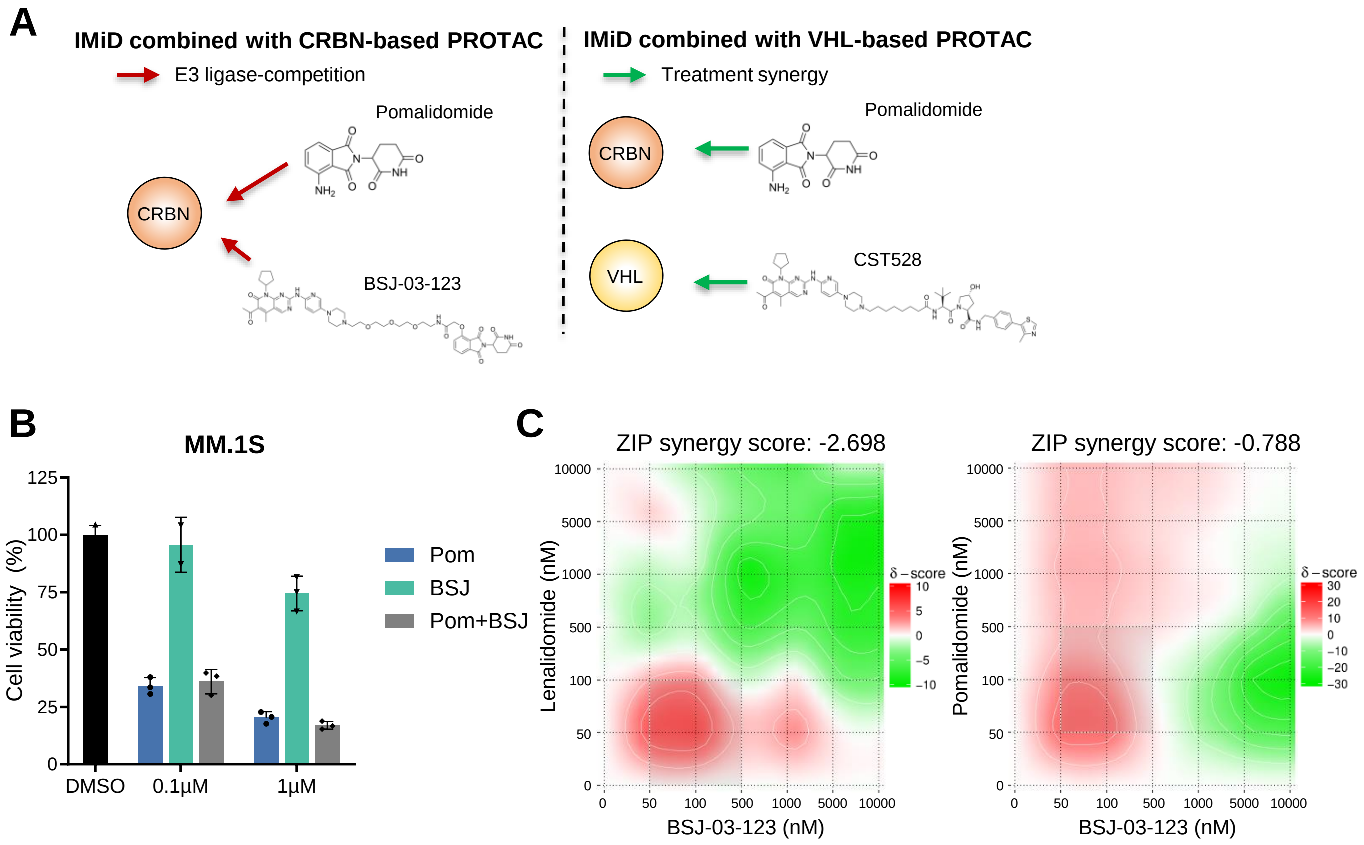

Supplementary Figure 9. Overcoming antagonism by CRBN-based PROTACs with VHL-based CDK6 PROTAC. (A) Schematic diagram of E3 ligase competition upon combination treatment of IMiD with CRBN-based PROTAC versus synergistic treatment with VHL-based PROTAC. (B) Cell viability of MM.1S after 96 h treatment of pomalidomide (pom), BSJ-03-123 (BSJ), or in combination (pom+BSJ). (N=3 biologically independent replicates) (C) Synergy map of MM.1S cells treated with BSJ-03-123 in combination with lenalidomide and pomalidomide. Synergy levels are indicated with ZIP synergy scores. Synergy maps were generated with SynergyFinder.[75] Viability data represent the mean ±SD of biological triplicates. Synergy data represent the mean of biological triplicates.

Supplementary Figure 10

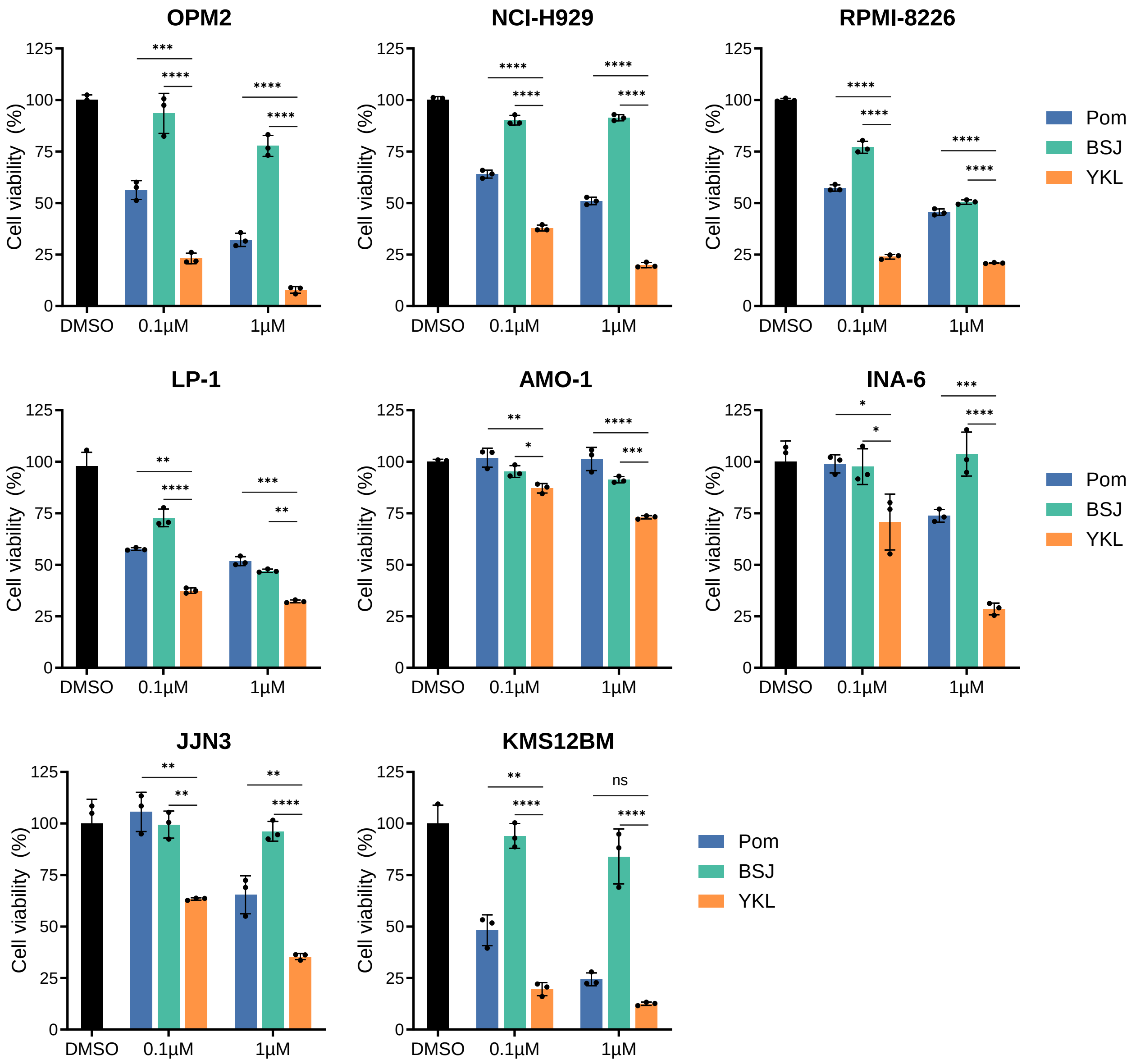

Supplementary Figure 10. YKL-06-102 shows high level of intramolecule synergism via CDK6, IKZF1, and IKZF3 degradation. Cell viability of IMiD-sensitive and -resistant multiple myeloma cell lines after 96 h treatment with pomalidomide (pom), BSJ-03-123 (BSJ), and YKL-06-102 (YKL). (N=3 biologically independent replicates) Cell viability is normalized to respective DMSO conditions. Data represent the mean ±SD of biological triplicates. One-way ANOVA is applied. P values are displayed as follows: n.s. = $P > 0.05$; * = $P \leq 0.05$; ** = $P \leq 0.01$; *** = $P \leq 0.001$; **** = $P \leq 0.0001$.

Supplementary Figure 11

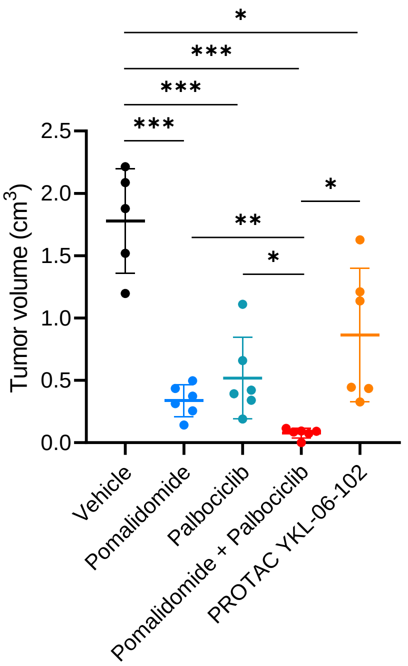

Supplementary Figure 11. *In vivo* effects of pomalidomide, CDK6 inhibition and CDK6/IKZF1/IKZF3-targeting PROTAC. Tumor size on day 9. Differences in tumor volumes were analyzed by unpaired t-tests with Welch's correction. Data represent the mean ±SD of biological replicates. Group size: n=5 for vehicle group; n=6 for pomalidomide treatment group; n=6 for palbociclib treatment group; n=6 for pomalidomide + palbociclib treatment group; n=6 for YKL-06-102 treatment group. P values are displayed as follows: * = $P \leq 0.05$; ** = $P \leq 0.01$; *** = $P \leq 0.001$.

Supplementary Figure 12

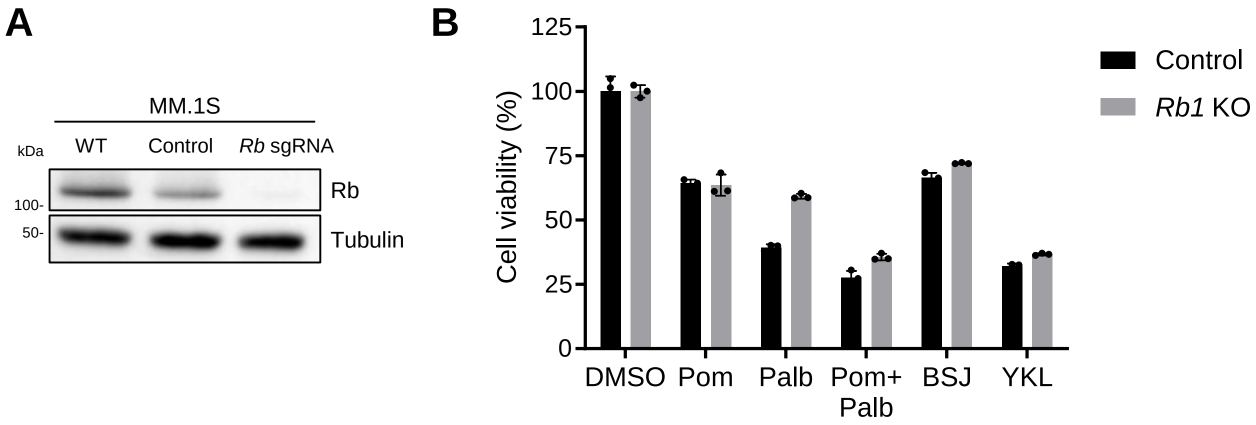

Supplementary Figure 12. Combination treatment is independent of *Rb1*. (A) Western blot analysis for confirmation of CRISPR/Cas9-mediated knockout of *Rb1* in MM.1S cells. (B) Cell viability of various single or combined treatments in MM.1S control or *Rb1* KO cells at 1 µM each. Cells were treated for 96 h with pomalidomide (pom), palbociclib (palb), in combination (pom+palb), BSJ-03-123 (BSJ), and YKL-06-102 (YKL). (N=3 biologically independent replicates) Control denotes Cas9- cells transduced with luciferase sgRNA. Cell viability is normalized to respective DMSO conditions. Data represent the mean ±SD of biological triplicates.

Supplementary Figure 13

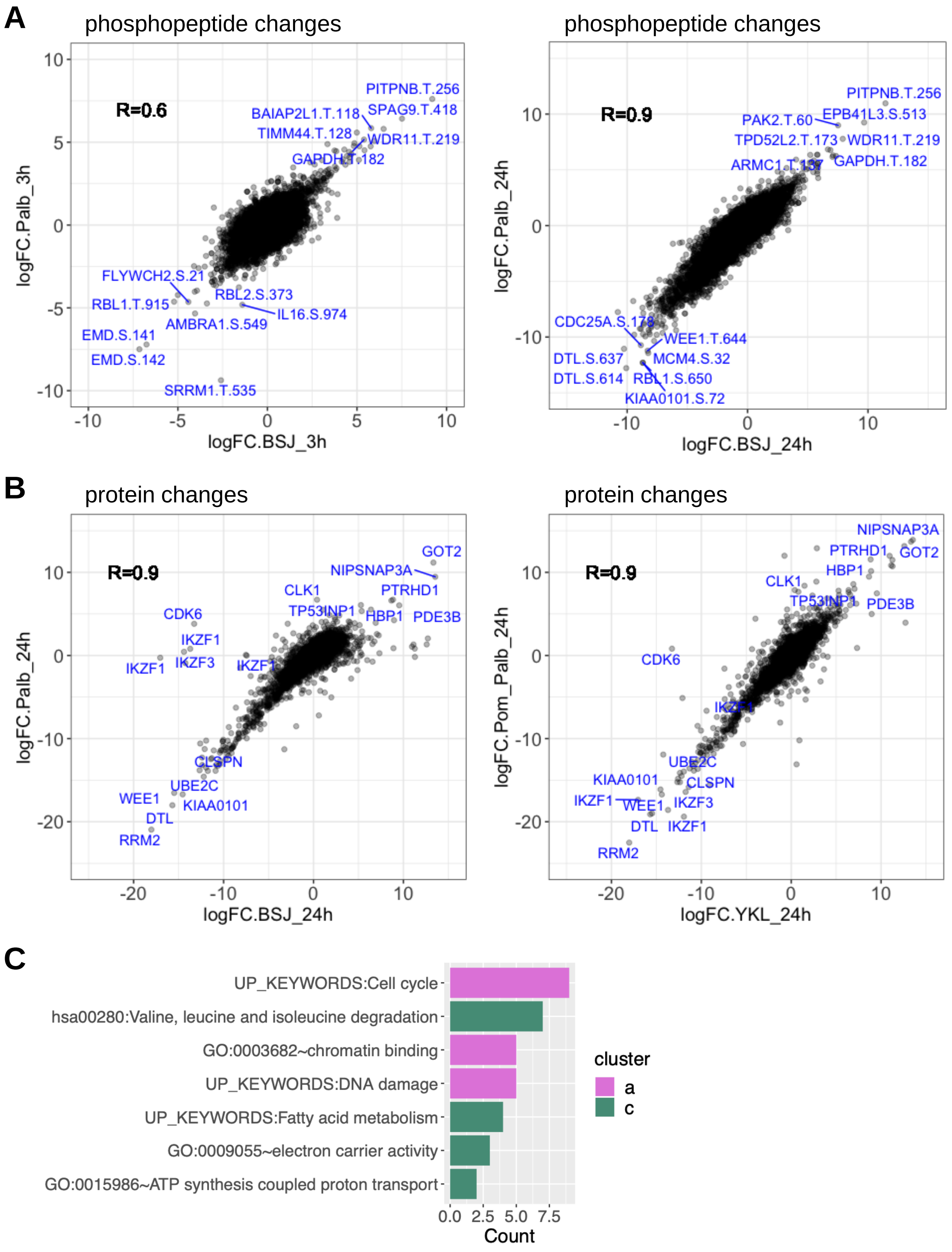

Supplementary Figure 13. Correlation of CDK6 inhibition by palbociclib and CDK6 degradation by PROTACs. MM.1S cells were treated for 3 h or 24 h as indicated. Dotplots show correlation of fold changes of (A) phosphopeptides or (B) proteins relative to control treated cells. (C) Representative GO annotation of proteins in cluster a and cluster c of Figure 6B. Pom = pomalidomide; Palb = palbociclib; BSJ = BSJ-03-123; YKL = YKL-06-102.

Supplementary Figure 14

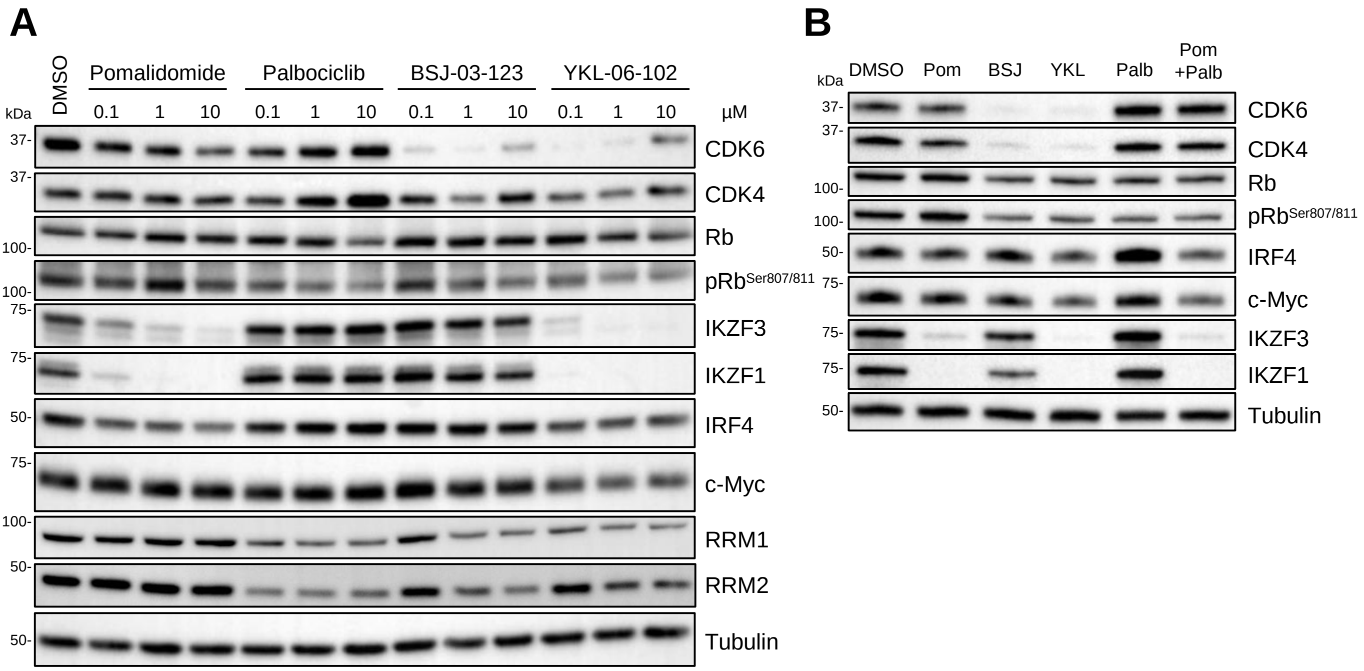

Supplementary Figure 14. Protein analyses of multiple myeloma cells treated with pomalidomide, CDK6 inhibitors and PROTACs. (A) Western blot analysis of OPM2 cells treated for 16 h with pomalidomide, palbociclib, BSJ-03-123, and YKL-06-102 at indicated concentrations. (N=3 biologically independent replicates) (B) Western blot analysis of MM.1S cells after subjected to respective drug treatments at 1 µM for 16 h. Cells were treated with pomalidomide (pom), palbociclib (palb), in combination (pom+palb), BSJ-03-123 (BSJ), and YKL-06-102 (YKL). (N=3 biologically independent replicates)

Supplementary Figure 15

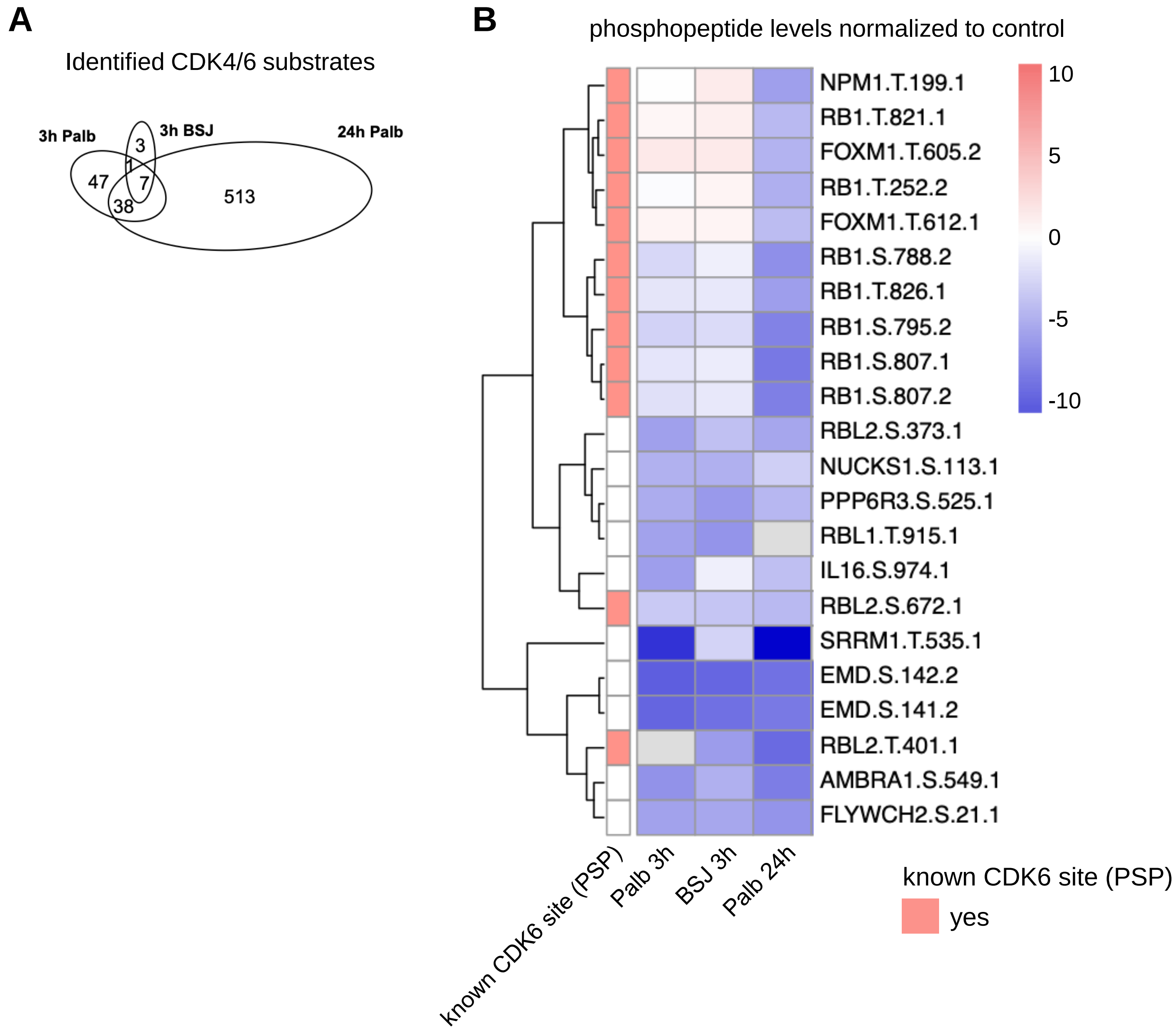

Supplementary Figure 15. Phosphohoproteomic analysis of drug-treated MM.1S cells identifies potential CDK4/6 substrates in multiple myeloma cells. (A) Overlap of putative CDK4/6 substrates in MM.1S cells. (B) Fold change relative to control of most regulated phosphopeptides and known CDK6 targets from phosphosite plus (PSP). Palb = palbociclib; BSJ = BSJ-03-123.

Supplementary Figure 2B

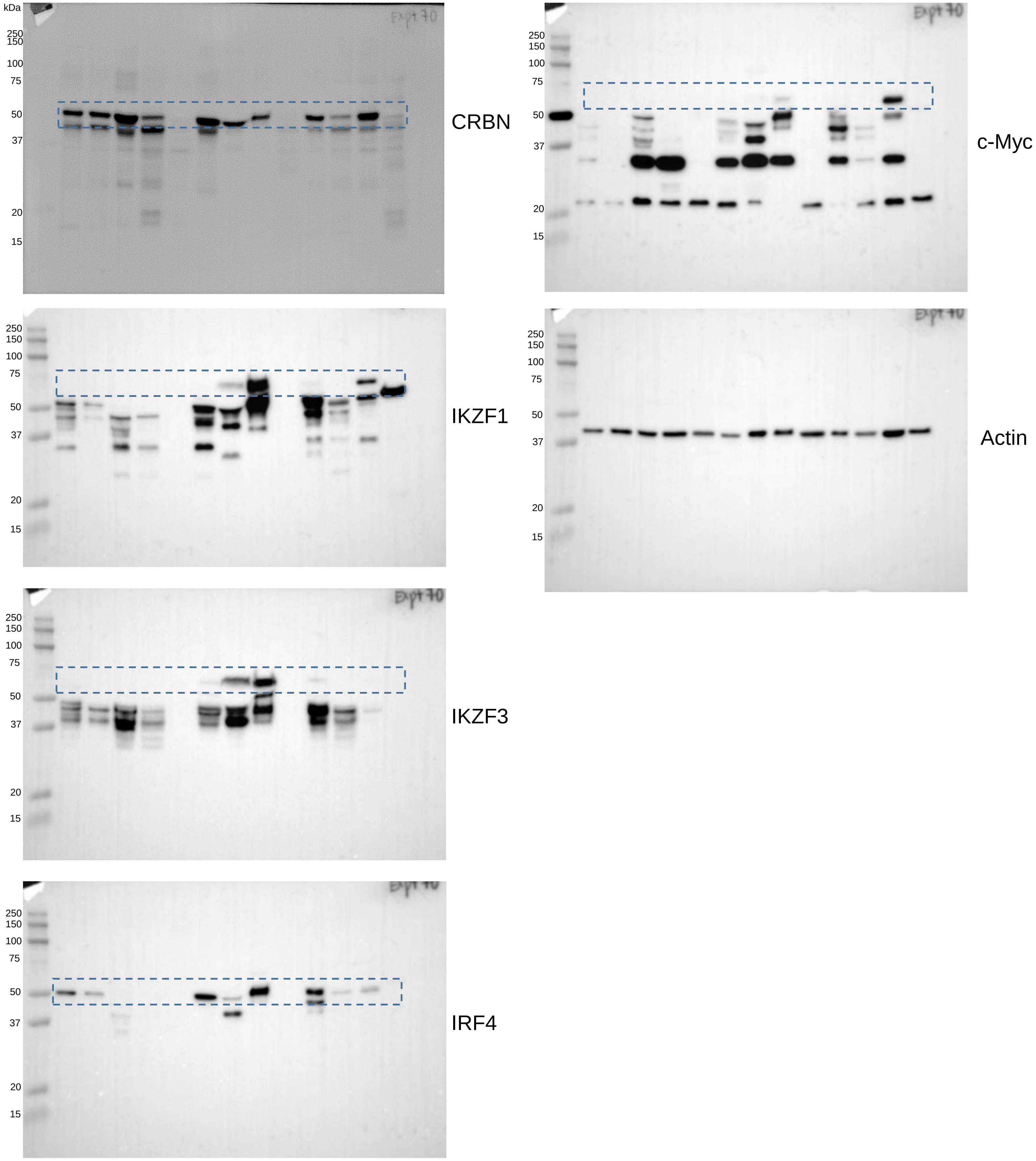

Supplementary Figure 2C

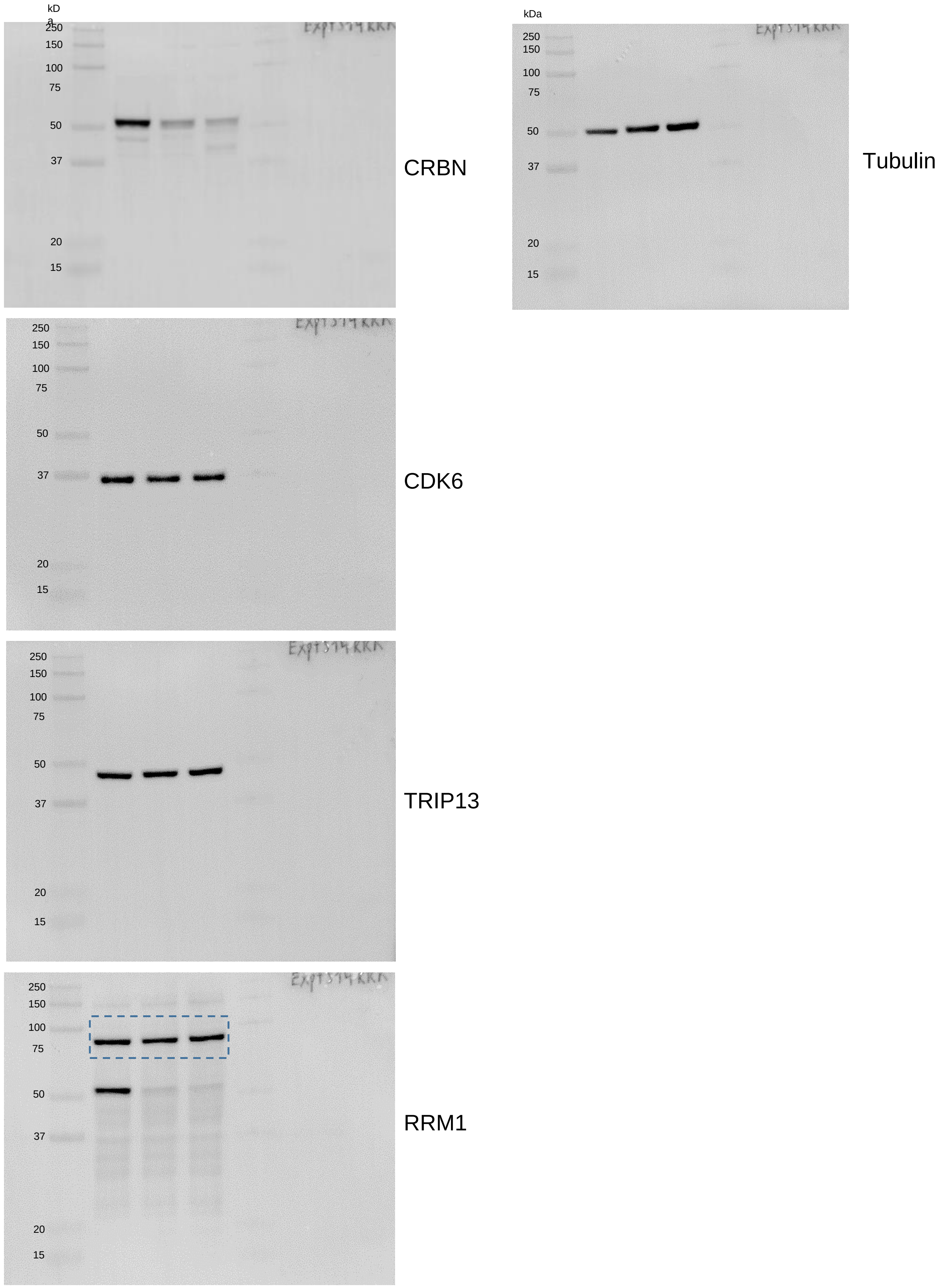

Supplementary Figure 4A

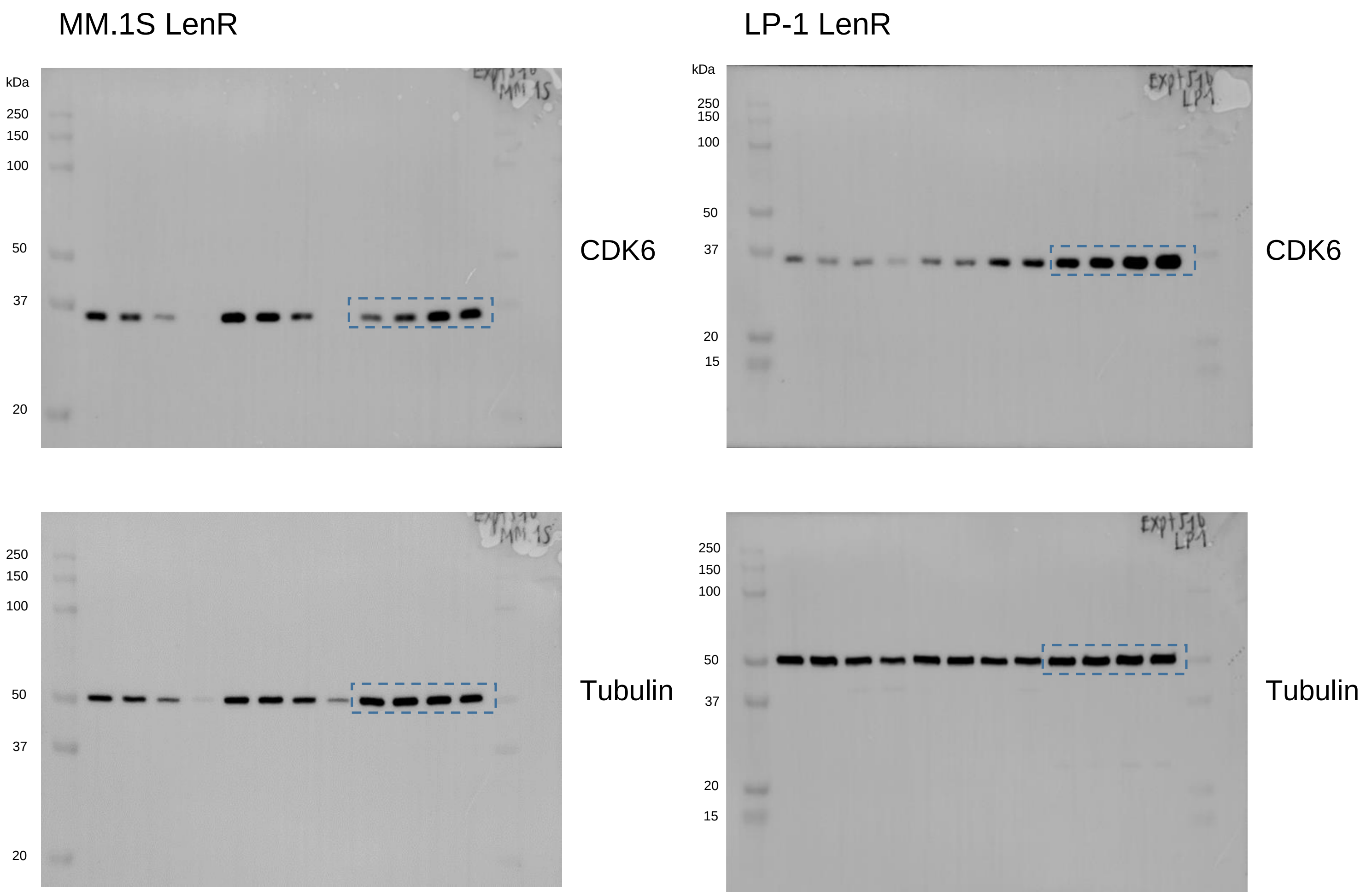

Supplementary Figure 4B

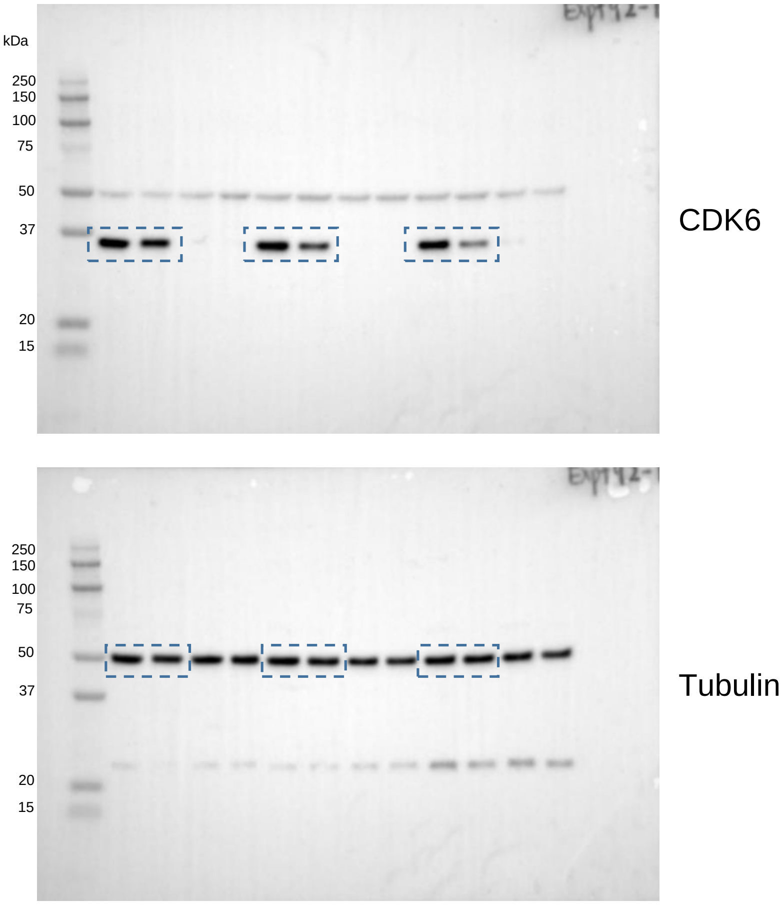

Supplementary Figure 4C

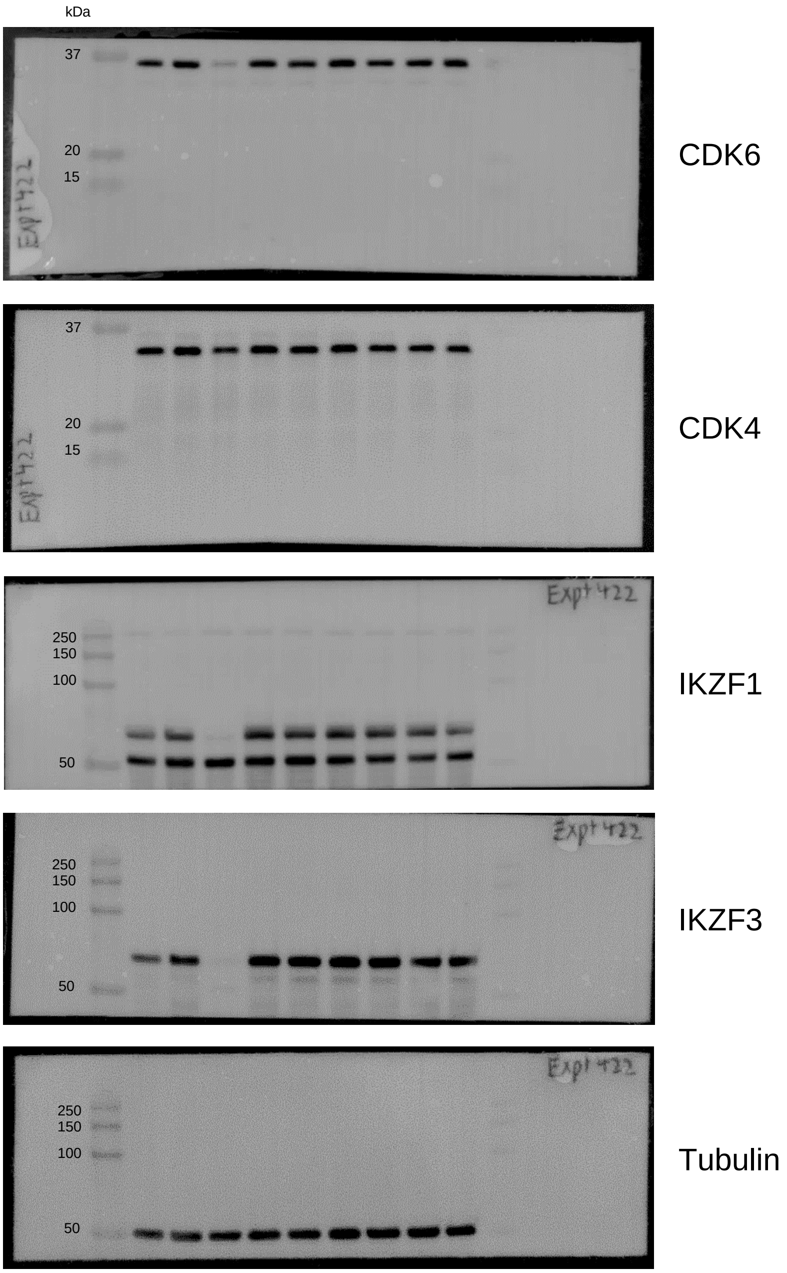

Supplementary Figure 5A

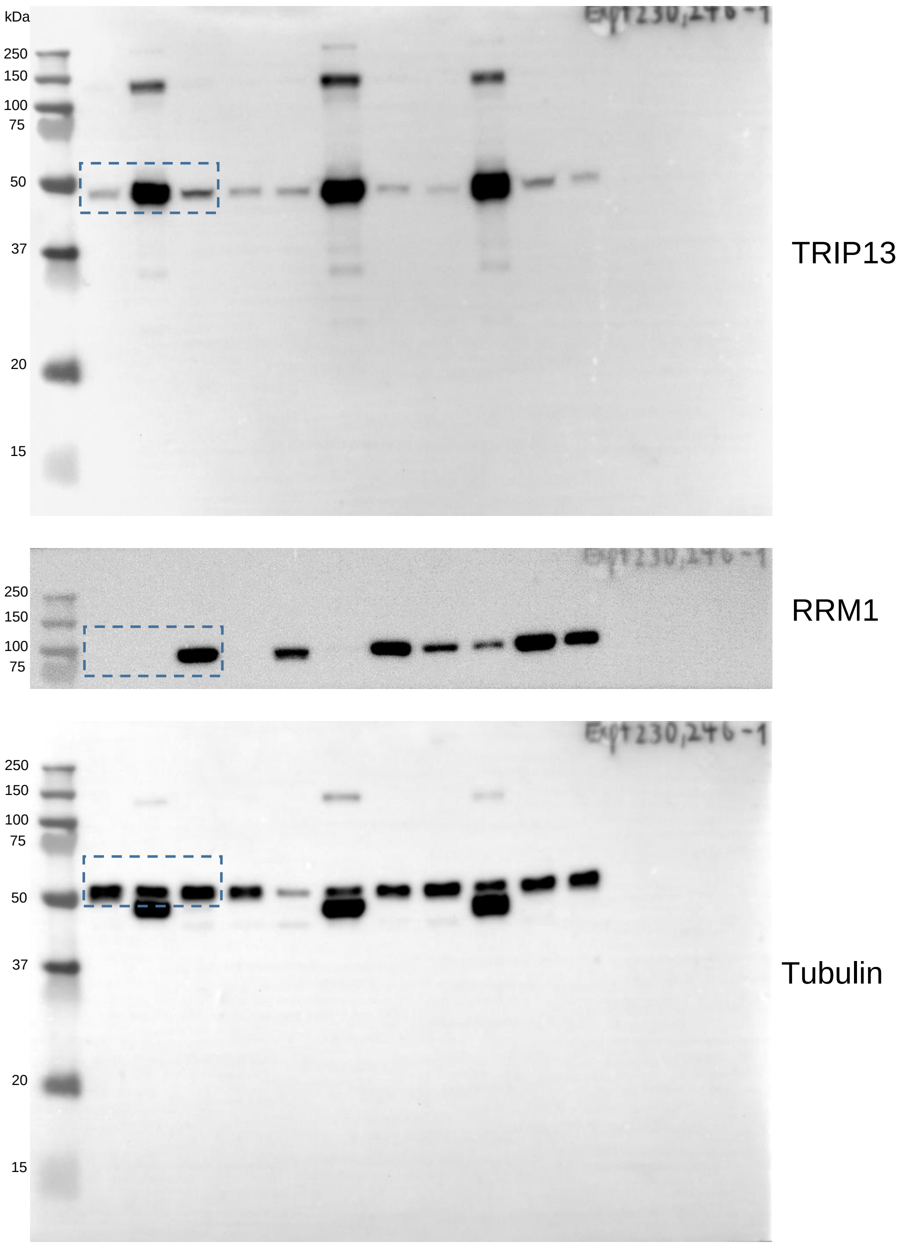

Supplementary Figure 7B

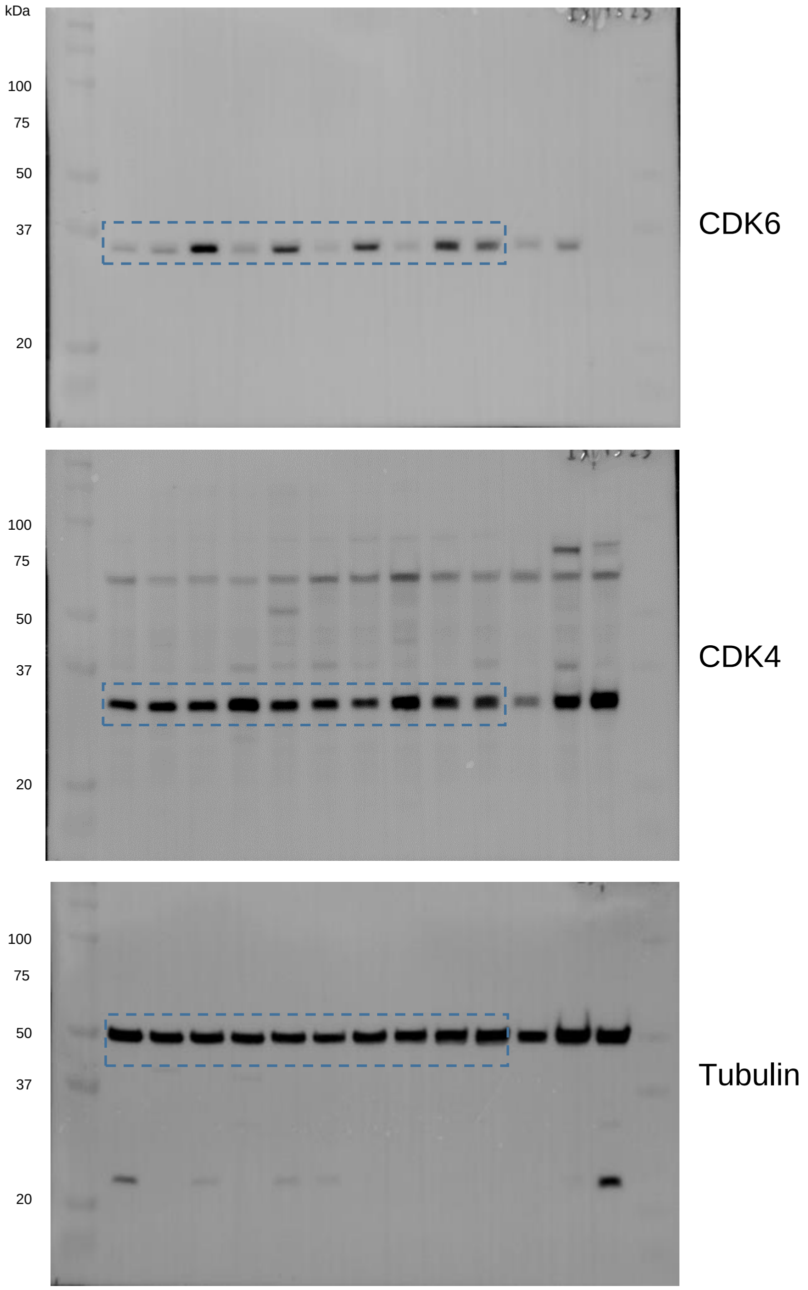

Supplementary Figure 12A

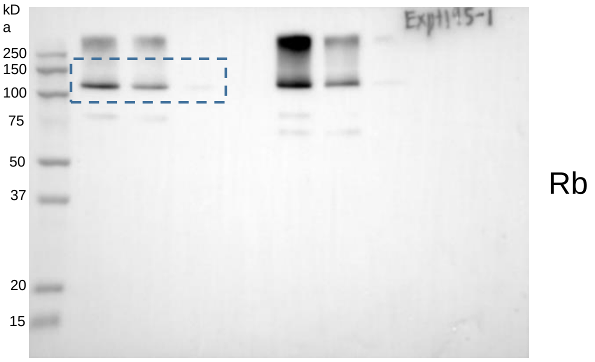

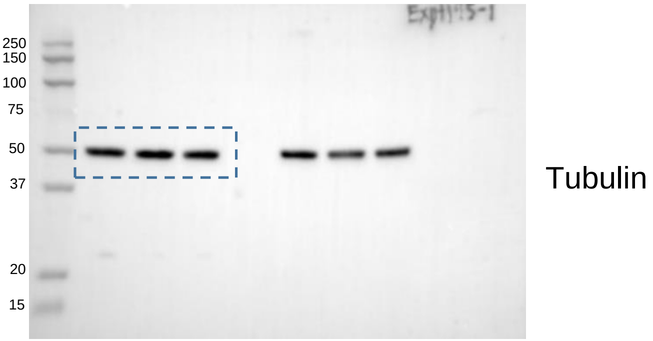

Supplementary Figure 14A

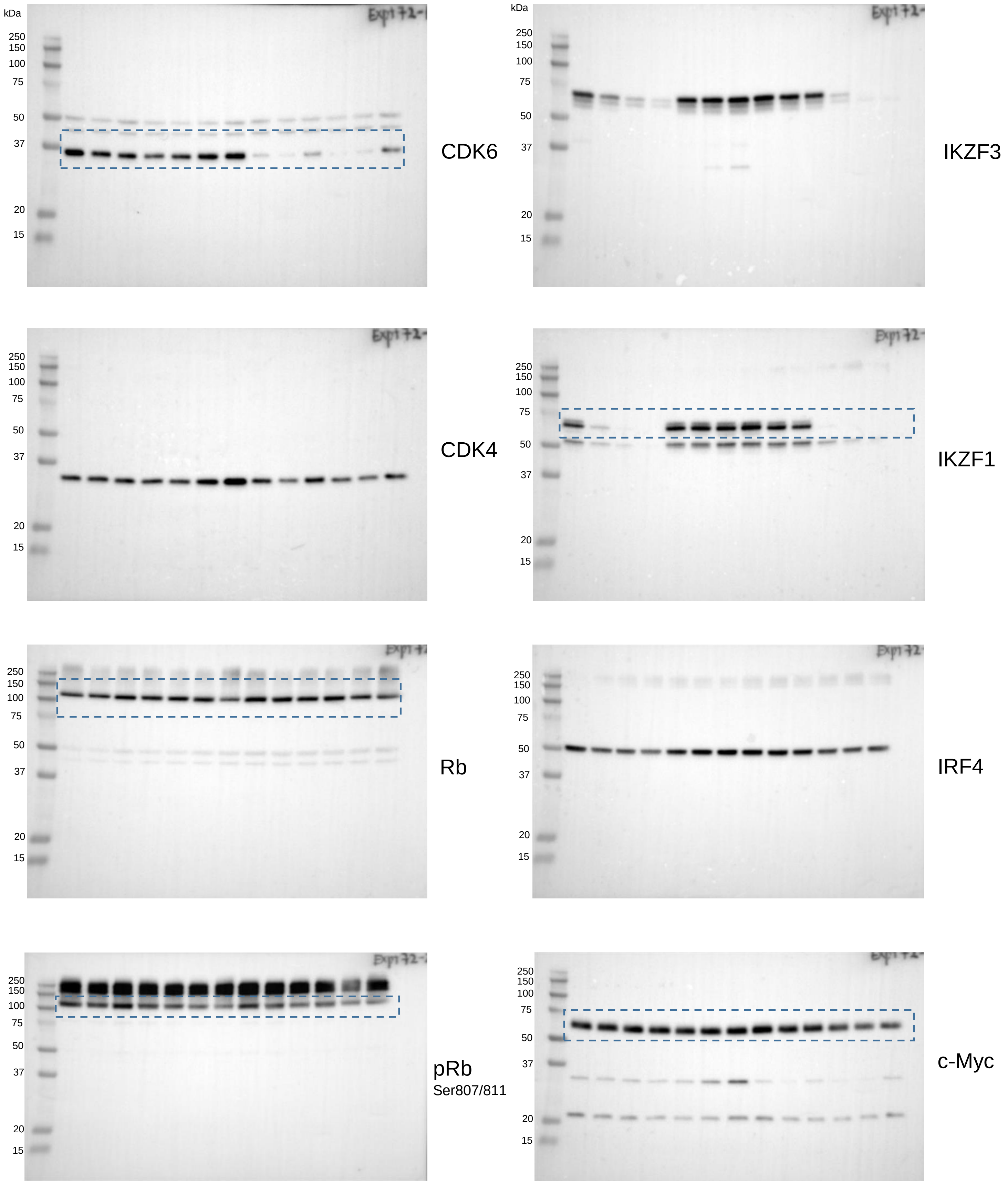

Supplementary Figure 14A

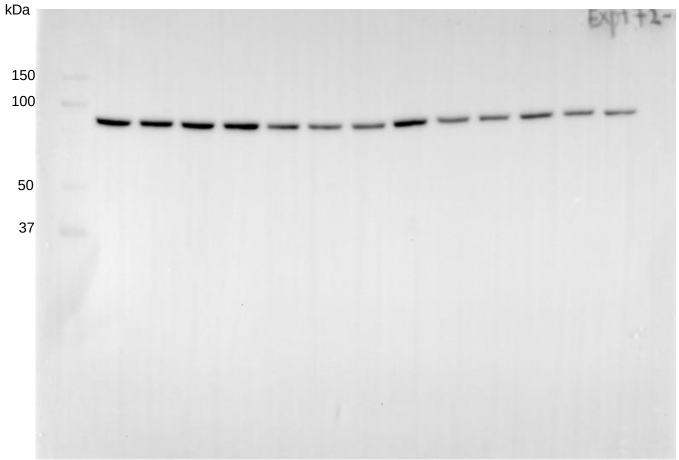

RRM1

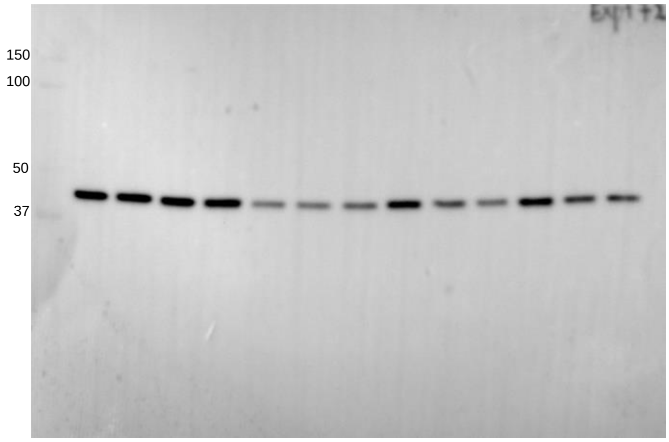

RRM2

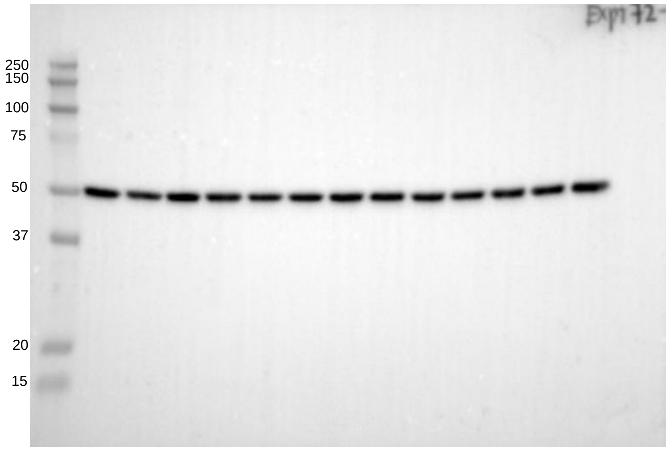

Tubulin

Supplementary Figure 14B

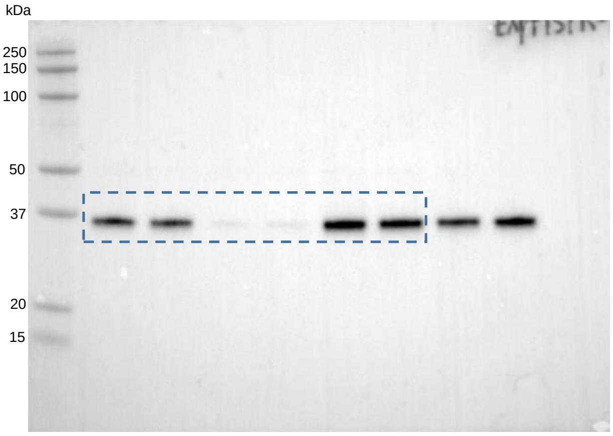

CDK6

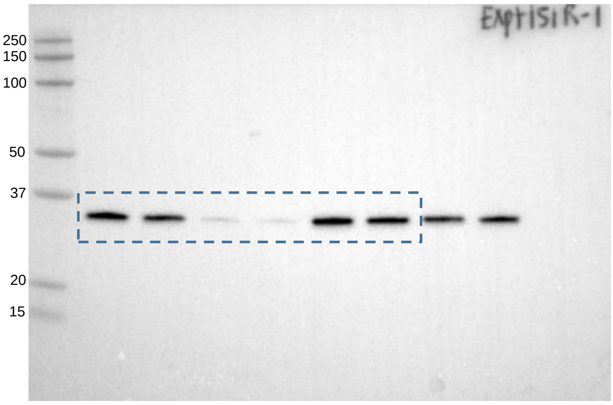

CDK4

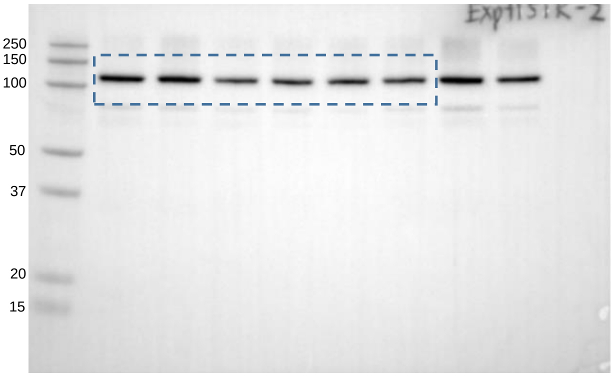

Rb

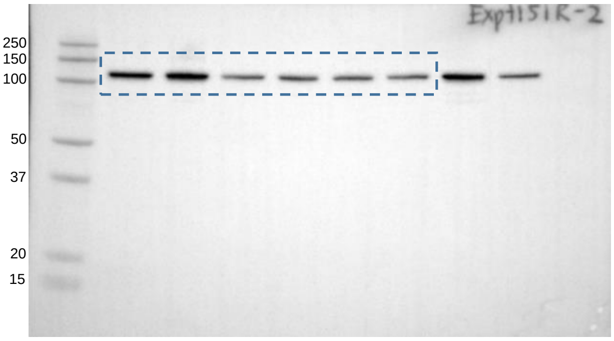

pRb$^{Ser807/811}$

Supplementary Figure 14B

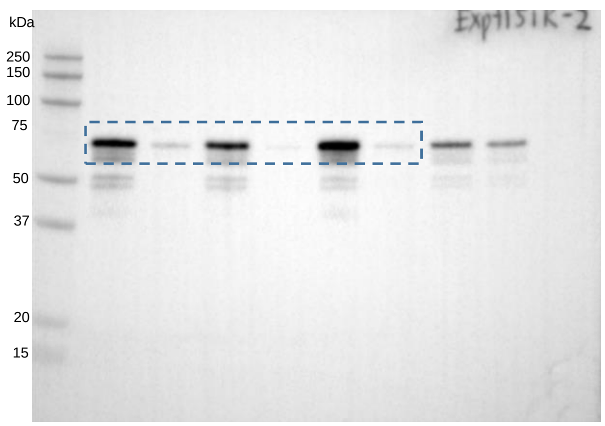

IKZF3

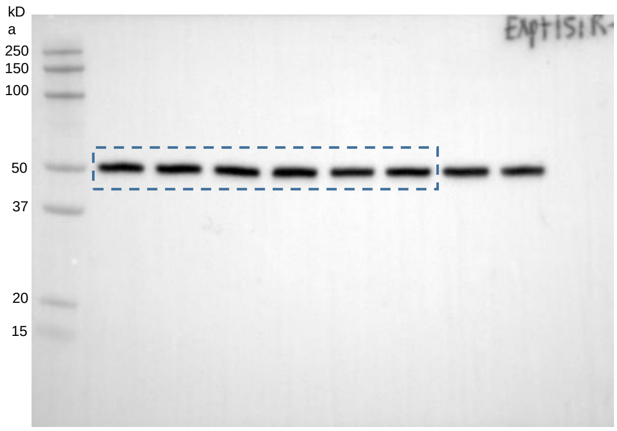

Tubulin

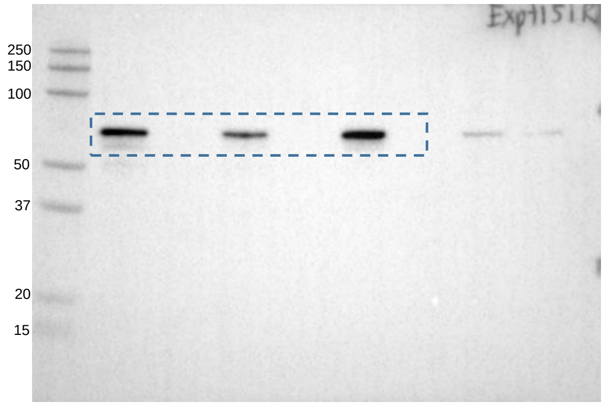

IKZF1

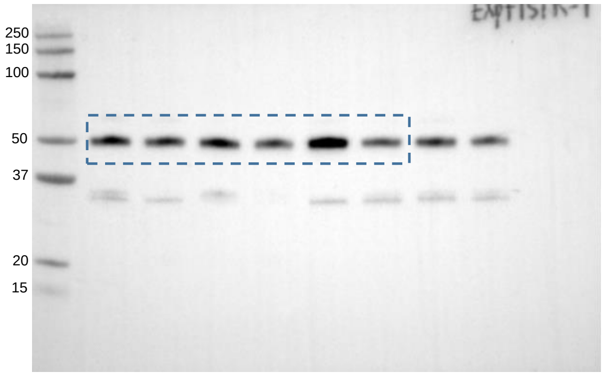

IRF4

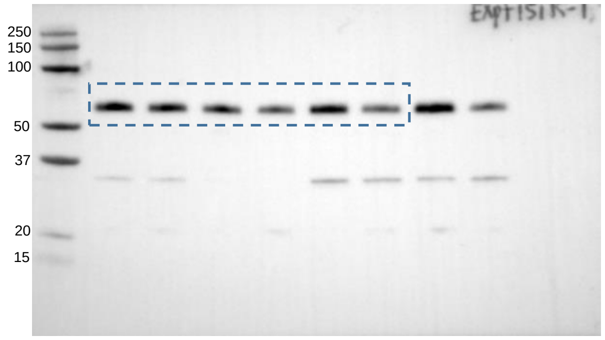

c-Myc
