# Supplementary material for: Proteomic profiling reveals CDK6 upregulation as a targetable resistance mechanism for lenalidomide in multiple myeloma
Source: Nat Commun. 2022 Feb 23;13:1009. doi: 10.1038/s41467-022-28515-1 (PMC8866544; doi:10.1038/s41467-022-28515-1)
Supplement: Supplementary file 3 — Description of Supplementary Datsets [file 41467_2022_28515_MOESM3_ESM.pdf]

Description of Additional Supplementary Files

File Name: Supplementary Data 1
Description: TMT proteomic and phosphoproteomic datasets of multiple myeloma patient samples
Sheet 1 (channel_annotation) contains TMT channel annotations for all datasets
Sheet 2 (columns) contains column annotations for sheet 3 and 4
Sheet 3 (patient_data_gloabal_plex1) contains global proteome data for multiple myeloma patient samples
Sheet 4 (patient_data_gloabal_plex1) contains phosphoproteome data for multiple myeloma patient samples

File Name: Supplementary Data 2
Description: RNAseq data of the same multiple myeloma patient samples that were also analyzed with proteomics. Gene level data of relapse to diagnosis ratios as well as log2(TPM) values and median normalized TPM values

File Name: Supplementary Data 3
Description: TMT phosphoproteomic and proteomic datasets of drug treated MM1S cells
Sheet 1 (channel_annotation) contains experimental conditions and TMT channel annotation
Sheet 2 (columns) contains column annotations for sheet 3 and 4
Sheet 3 (MM1S_globalproteome) contains global proteome data
Sheet 4 (MM1S_gphosphoproteome) contains phosphoproteome data

File Name: Supplementary Data 4
Description: CDK6 relapse signature
Contains proteins significantly regulated at relapse and their corresponding levels in patient samples and drug treated MM1S cells (related to Figure 6B)
