# Supplementary material for: Proteomic profiling reveals CDK6 upregulation as a targetable resistance mechanism for lenalidomide in multiple myeloma
Source: Nat Commun. 2022 Feb 23;13:1009. doi: 10.1038/s41467-022-28515-1 (PMC8866544; doi:10.1038/s41467-022-28515-1)
Supplement: Supplementary file 8 — Reporting Summary [file 41467_2022_28515_MOESM8_ESM.pdf]

nature research

Corresponding author(s): Jan Krönke
Philipp Mertins

Last updated by author(s): Dec 30, 2021

Reporting Summary

Nature Research wishes to improve the reproducibility of the work that we publish. This form provides structure for consistency and transparency in reporting. For further information on Nature Research policies, see our Editorial Policies and the Editorial Policy Checklist.

Statistics

For all statistical analyses, confirm that the following items are present in the figure legend, table legend, main text, or Methods section.

n/a	Confirmed	
☐	☒	The exact sample size (*n*) for each experimental group/condition, given as a discrete number and unit of measurement
☐	☒	A statement on whether measurements were taken from distinct samples or whether the same sample was measured repeatedly
☐	☒	The statistical test(s) used AND whether they are one- or two-sided<br>*Only common tests should be described solely by name; describe more complex techniques in the Methods section.*
☒	☐	A description of all covariates tested
☐	☒	A description of any assumptions or corrections, such as tests of normality and adjustment for multiple comparisons
☐	☒	A full description of the statistical parameters including central tendency (e.g. means) or other basic estimates (e.g. regression coefficient) AND variation (e.g. standard deviation) or associated estimates of uncertainty (e.g. confidence intervals)
☐	☒	For null hypothesis testing, the test statistic (e.g. F, t, r) with confidence intervals, effect sizes, degrees of freedom and P value noted<br>*Give P values as exact values whenever suitable.*
☒	☐	For Bayesian analysis, information on the choice of priors and Markov chain Monte Carlo settings
☒	☐	For hierarchical and complex designs, identification of the appropriate level for tests and full reporting of outcomes
☐	☒	Estimates of effect sizes (e.g. Cohen's *d*, Pearson's *r*), indicating how they were calculated

Our web collection on statistics for biologists contains articles on many of the points above.

Software and code

Policy information about availability of computer code

Data collection	Proteomics raw data was collected on a Q Exactive HF-X mass sepctrometer with Thermo Xcalibur software version 4.1.31.9<br>Single end RNAseq raw data was collected on a GPL11154 HiSeq 2000 platform with HiSeq Control Software (HSC) 2.2.68<br>Cell viability data was collected on POLARstar® Omega plate reader with Ω MARS Data Analysis Software version 1.11<br>Western blot images were collected with ChemiDoc™ XRS+ System with BioRad ImageLab version 5.2.1; and Fujifilm Luminescent Image Analyzer LAS-4000mini with Image Reader LAS-4000mini software and Multi Gauge software version3.1<br>qRT-PCR data was collected with StepOnePlus Real-Time PCR System and StepOnePlus Software version 2.3
Data analysis	Proteomics: raw data search with MaxQuant Version 1.6.3.3<br>RNAseq: alignment with STAR 2.7, quantification with RSEM 1.3.0<br>Further analysis of proteomics and RNAseq data was performed with R(4.0.3) and R studio (Version 1.3.1093). Moderated one-sample t-test was performed with the limma package integrated in the ProTIGY application provided by the Broad institute on GitHub (https://github.com/broadinstitute/protigy), single sample gene set enrichment analysis was performed with ssGSEA 2.0 tool (https://github.com/broadinstitute/ssGSEA2.0), GO enrichment was perfromed with the online DAVID tool v6.8<br>Calculation and statistics: Microsoft Excel 2016, GraphPad Prism v8 and v9.1.0

For manuscripts utilizing custom algorithms or software that are central to the research but not yet described in published literature, software must be made available to editors and reviewers. We strongly encourage code deposition in a community repository (e.g. GitHub). See the Nature Research guidelines for submitting code & software for further information.

Data

Policy information about availability of data

All manuscripts must include a data availability statement. This statement should provide the following information, where applicable:

- Accession codes, unique identifiers, or web links for publicly available datasets
- A list of figures that have associated raw data
- A description of any restrictions on data availability

The mass spectrometry proteomics data and search results generated in this study have been deposited to the ProteomeXchange Consortium via the PRIDE partner repository70 with the dataset identifier PXD021265 (https://www.ebi.ac.uk/pride/archive/projects/PXD021265). The human reference proteome (UP000005640) was downloaded from UniProt in 01/2017 (https://ftp.uniprot.org/pub/databases/uniprot/previous_releases/). The RNA-sequencing data generated in this study are available on Gene Expression Omnibus under accession number GSE162403 (https://www.ncbi.nlm.nih.gov/geo/query/acc.cgi?acc=GSE162403). Processed proteomics data are available in Supplementary Data 1 (patient proteomics data) and Supplementary Data 3 (MM1S proteomics data); processed patient RNAseq data is available in Supplementary Data 2. Source data are provided with this paper. The remaining data are available within the Article, Supplementary Information or Source Data file.

Field-specific reporting

Please select the one below that is the best fit for your research. If you are not sure, read the appropriate sections before making your selection.

[x] Life sciences [ ] Behavioural & social sciences [ ] Ecological, evolutionary & environmental sciences

For a reference copy of the document with all sections, see nature.com/documents/nr-reporting-summary-flat.pdf

Life sciences study design

All studies must disclose on these points even when the disclosure is negative.

Sample size	For proteomic analysis of patient samples, sample size was chosen based on number of available TMT channels (TMT10-plex) at the beginning of the study<br>For proteomic analysis of cell culture samples, we previously observed that biological duplicates are sufficent for meaningful data generation (Krönke et al, Science 2014). Here, we confirm high reproducibility of proteomic analysis of cell culture samples.<br>For the in vivo study the effect size of treatment response of xenografted myeloma model to palbociclib was calculated with G'Power (v3.1.9.7; University Düsseldorf, Germany) and a sample size of n=6 animals per group was determined for a power of 80% for the animal experiment in this study.
Data exclusions	Due to limited protein amounts of the patient 5 diagnosis sample, patient 5 was excluded from the analysis of the phosphoproteomic data.<br>In the in vivo study, no data were excluded.
Replication	Top targets from proteomic analysis of patient samples were validated by western blot analysis of an independent cohort of non-paired samples. Proteomic changes detected in cell lines were validated via western blot in at least 3 independent experiments.<br>Cell viability and western blot analyses were replicated in at least 2 independent experiments.<br>The in vivo study was performed once and analyses were performed regarding changes in tumor growth and animal survival.
Randomization	Retrospective patient samples for proteomic analysis were chosen based on sample availability and treatment regimen and no randomization (no allocation of treatment groups) occured. TMT channels were randomly assigned.<br>Before start of treatments, tumor bearing mice were randomized and allocated to the various treatment groups with 6 mice per group.
Blinding	Retrospective patient samples for proteomic analysis were chosen based on sample availability and treatment regimen and no blinding (no allocation of treatment groups) occured. TMT channels were randomly assigned.<br>For the in vivo study groups were labeled with group A to E without detailed specification of the respective drug for treatment of the groups during the course of the study.

Reporting for specific materials, systems and methods

We require information from authors about some types of materials, experimental systems and methods used in many studies. Here, indicate whether each material, system or method listed is relevant to your study. If you are not sure if a list item applies to your research, read the appropriate section before selecting a response.

Materials & experimental systems

n/a	Involved in the study
☐	☒ Antibodies
☐	☒ Eukaryotic cell lines
☒	☐ Palaeontology and archaeology
☐	☒ Animals and other organisms
☐	☒ Human research participants
☒	☐ Clinical data
☒	☐ Dual use research of concern

Methods

n/a	Involved in the study
☒	☐ ChIP-seq
☒	☐ Flow cytometry
☒	☐ MRI-based neuroimaging

Antibodies

Antibodies used

Primary antibodies used for Western blotting from Cell Signaling (Danvers, USA) include CDK6 (clone DCS83, #3136, , RRID:AB_2229289, 1:2000), CDK4 (clone D9G3E, #12790, RRID:AB_2631166, 1:1000), Rb (clone 4H1, #9309, RRID:AB_823629, 1:1000), Phospho-Rb (Ser807/811) (#9308, RRID:AB_331472, 1:1000), IKZF3 (clone D1C1E, #15103, RRID:AB_2744524, 1:1000), IKZF1 (clone D6N9Y, #14859, RRID:AB_2744523, 1:1000), IRF4 (clone D43H10, #4299, RRID:AB_10547141, 1:1000), c-Myc (clone D84C12, #5605, RRID:AB_1903938, 1:1000), RRM1 (clone D12F12, #8637, RRID:AB_11217623, 1:1000), RRM2 (clone E7Y9J, #65939, 1:1000), anti-rabbit IgG HRP-linked antibody (#7074, 1:5000), anti-mouse IgG HRP-linked antibody (#7076, 1:5000); antibodies from Sigma-Aldrich (St. Louis, USA) include anti-α-Tubulin (#T5168, RRID:AB_477579, 1:7000); antibodies from Santa Cruz Biotechnology (Dallas, USA) include CDK6 (clone B-10, sc-7961, 1:1000), TRIP13 (clone A-7, sc-514285, 1:1000); antibodies from Abcam (Cambridge, United Kingdom) include anti-β-actin (ab20272, 1:10,000).

Validation

All primary antibodies were validated by respective manufacturer. RRID provided if available.

Eukaryotic cell lines

Policy information about cell lines

Cell line source(s)

All cell lines were purchased from DSMZ and/or ATCC.

Authentication

Authentification were performed by DSMZ. Authentification was performed on cell lines via STR profiling.

Mycoplasma contamination

All cell lines were tested negative for Mycoplasma contamination.

Commonly misidentified lines (See ICLAC register)

No commonly misidentified cell lines were used.

Animals and other organisms

Policy information about studies involving animals; ARRIVE guidelines recommended for reporting animal research

Laboratory animals

For the study, 6 - 8 week old female NOG mice were used (strain: NOD.Cg-Prkdcscid Il2rgtm1Sug/JicTac),). Mice for the study were obtained from Taconic (Leverkusen, Germany). The in vivo experiments were performed at EPO GmbH Berlin, Germany. The animals were group-housed (max. 5 mice/cage) in individually ventilated cages (IVC type GM 500, Techniplast) at temperature of 22°C±2°C, humidity of 50%±10%, at 12 h dark-light cycles.

Wild animals

This study did not involve wild animals

Field-collected samples

This study did not involve samples collected from the field

Ethics oversight

Animal study was performed at AAALAC-certified EPO GmbH, Berlin, Germany and has been approved and authorized by the Landesamt für Gesundheit und Soziales, Berlin, Germany (approval No. G 0333/18)

Note that full information on the approval of the study protocol must also be provided in the manuscript.

Human research participants

Policy information about studies involving human research participants

Population characteristics

All patient characteristics are given in Supplementary Figure 1.

Recruitment

This is a retrospective study. Samples were chosen from the Ulm University Multiple Myeloma biobank based on 1) informed consent from the patient, and 2) sufficient number and fraction of plasma cells without or after CD138+ selection. For samples analyzed by additional requirements were 3) availability of longitudinal samples and 4) treatment with lenalidomide (monotherapy or combination) between the two samples.

Ethics oversight

The study was approved by the internal review board of Ulm University.

Note that full information on the approval of the study protocol must also be provided in the manuscript.
